# Supplementary material for: Patient-derived tumor organoid and fibroblast assembloid models for interrogation of the tumor microenvironment in esophageal adenocarcinoma
Source: Cell Rep Methods. 2024 Nov 27;4(12):100909. doi: 10.1016/j.crmeth.2024.100909 (PMC11704619; doi:10.1016/j.crmeth.2024.100909)
Supplement: Document S1. Figures S1–S3 [file mmc1.pdf]

Cell Reports Methods, Volume *4*

Supplemental information

Patient-derived tumor organoid and fibroblast assembloid models for interrogation of the tumor microenvironment in esophageal adenocarcinoma

Benjamin P. Sharpe, Liliya A. Nazlamova, Carmen Tse, David A. Johnston, Jaya Thomas, Rhianna Blyth, Oliver J. Pickering, Ben Grace, Jack Harrington, Rushda Rajak, Matthew Rose-Zerilli, Zoe S. Walters, and Tim J. Underwood

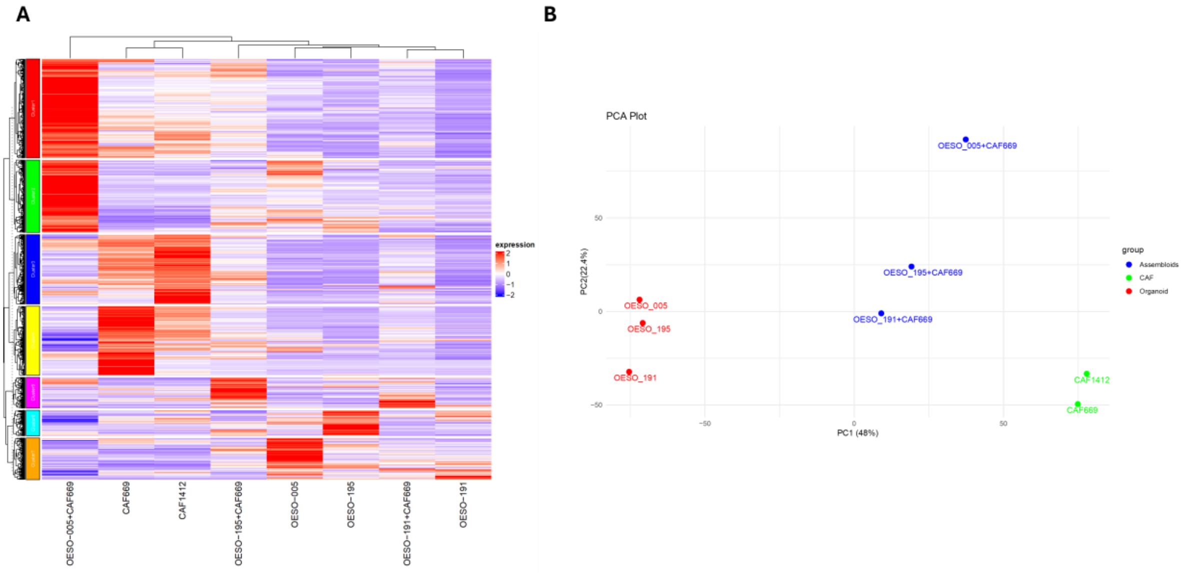

Figure S1. Descriptive analysis of RNA-seq data from assembloids and corresponding organoids and fibroblasts, related to Figure 2.

A.) To identify differences/similarities between assembloids and constituent components (organoids and CAFS), we performed cluster analysis on the top 2000 variable genes in the Transcript Per Millions count matrix to explore differences between samples. This analysis clusters the two CAF together and identified 7 distinct gene clusters, each showing unique gene expression patterns compared to the others. B.) To understand the distribution of samples, we conducted a PCA analysis revealing similarity across similar groups.

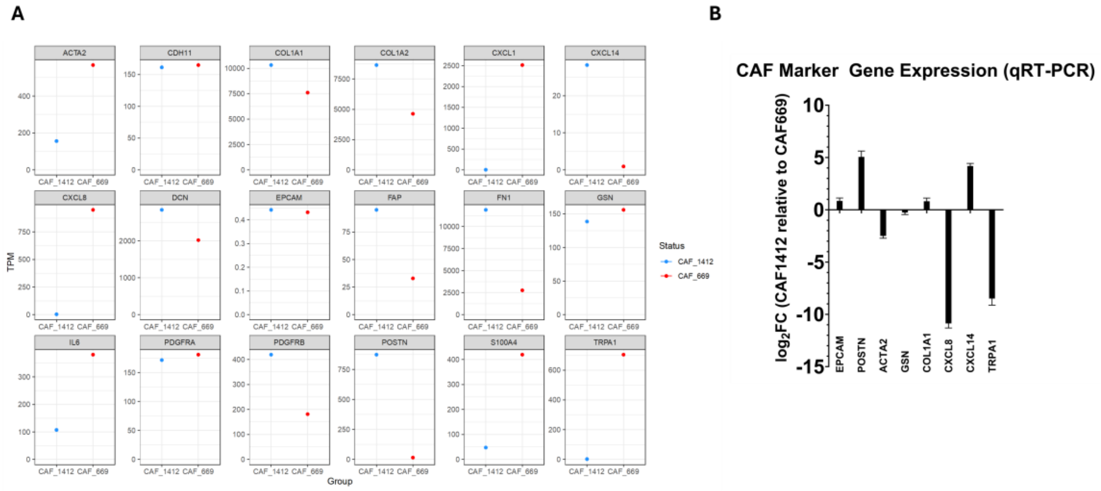

Figure S2. Phenotyping of CAF cultures by RNA-seq and qRT-PCR, related to Figure 2.

A.) Analysis of key myofibroblast and inflammatory CAF markers from bulk RNAseq data shows that both CAFs express key markers associated with myoCAF identity. CAF669 also expresses markers associated with a more proinflammatory CAF phenotype (IL6). B.) However, qRT-PCR analysis of key markers showed that the two CAFs were more similar in expression of myoCAF markers rather than in iCAF markers.

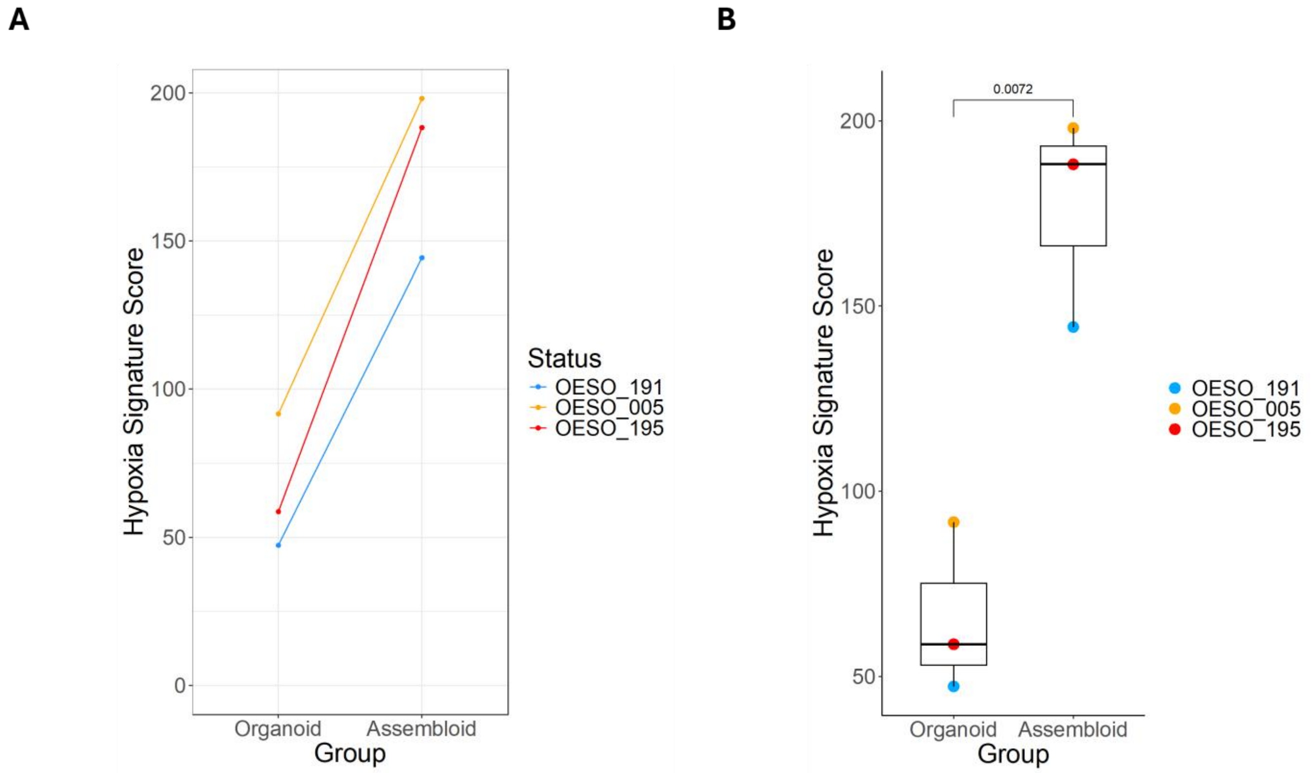

Figure S3. Comparison of hypoxia-related gene expression in organoids and corresponding assembloids, related to Figure 2.

A.) To identify whether hypoxia is a feature of co-cultures compared to organoids alone, we used the 200 genes in the hallmark hypoxia pathway to score each sample. B.) Analysis of bulk RNAseq data (n=3 sequenced samples per group) shows that in all cases the hypoxia signature is higher in co-culture, and when taken together the difference is significant (t-test).
