# Supplementary material for: Patient-derived tumor organoid and fibroblast assembloid models for interrogation of the tumor microenvironment in esophageal adenocarcinoma
Source: Cell Rep Methods. 2024 Nov 27;4(12):100909. doi: 10.1016/j.crmeth.2024.100909 (PMC11704619; doi:10.1016/j.crmeth.2024.100909)
Supplement: Document S2. Article plus supplemental information [file mmc6.pdf]

Cell Reports
Methods

Report

Patient-derived tumor organoid and fibroblast assembloid models for interrogation of the tumor microenvironment in esophageal adenocarcinoma

Graphical abstract

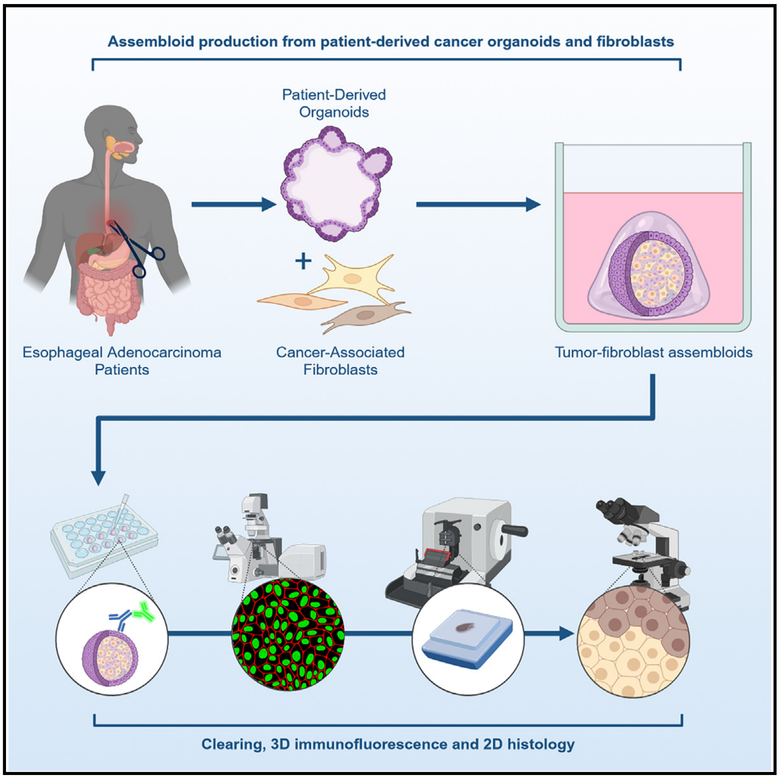

Highlights

- 3D assembloid models of esophageal tumor organoid-fibroblast crosstalk are presented
- Assembloids show similar histology to parental tumors
- Whole-mount immunofluorescence methods visualize tumor-stromal interactions in 3D
- Assembloids can be compared to parental organoids and fibroblasts using RNA-seq

Authors

Benjamin P. Sharpe, Liliya A. Nazlamova, Carmen Tse, ..., Matthew Rose-Zerilli, Zoe S. Walters, Tim J. Underwood

Correspondence

tju@soton.ac.uk

In brief

Tumor organoids model cancer heterogeneity, but non-cancer cell types are absent. Sharpe et al. present a method for producing assembloid cultures, combining tumor organoids and cancer-associated fibroblasts to model tumor-stromal interactions in 3D. They present a toolkit for characterizing these interactions using whole-mount immunofluorescence and histology.

Sharpe et al., 2024, Cell Reports Methods *4*, 100909
December 16, 2024 © 2024 The Author(s). Published by Elsevier Inc.
https://doi.org/10.1016/j.crmeth.2024.100909

CellPress

Cell Reports Methods

CellPress
OPEN ACCESS

Report

Patient-derived tumor organoid and fibroblast assembloid models for interrogation of the tumor microenvironment in esophageal adenocarcinoma

Benjamin P. Sharpe,[1] Liliya A. Nazlamova,[1,2] Carmen Tse,[1] David A. Johnston,[3] Jaya Thomas,[4] Rhianna Blyth,[1] Oliver J. Pickering,[1,5] Ben Grace,[1,6] Jack Harrington,[1] Rushda Rajak,[5] Matthew Rose-Zerilli,[1] Zoe S. Walters,[1] and Tim J. Underwood[1,4,7,*]

[1]School of Cancer Sciences, Faculty of Medicine, University of Southampton, Southampton SO16 6YD, UK
[2]Early Cancer Institute, University of Cambridge, Department of Oncology, Box 197 Cambridge Biomedical Campus, Hills Road, Cambridge SB2 0XZ, UK
[3]Biomedical Imaging Unit, Faculty of Medicine, University of Southampton, Southampton SO16 6YD, UK
[4]Bio-R Bioinformatics Research Facility, Faculty of Medicine, University of Southampton, Southampton SO16 6YD, UK
[5]University Hospital Southampton NHS Foundation Trust, Southampton SO16 6YD, UK
[6]Southampton Clinical Trials Unit, University of Southampton, Southampton, UK
[7]Lead contact
*Correspondence: tju@soton.ac.uk
https://doi.org/10.1016/j.crmeth.2024.100909

MOTIVATION Patient-derived organoids (PDOs) retain more three-dimensional (3D) tissue-like complexity than traditional 2D cell line models, and they are increasingly used to understand cancer biology and model the effects of cancer treatments. Conventional PDOs only include the tumor cell compartment, and consequently, crucial tumor microenvironment (TME) interactions that shape cancer biology, such as contact with stromal and immune cells, are absent. We aimed to develop a robust approach for producing 3D assembloid cultures to model the TME in esophageal adenocarcinoma (EAC) by incorporating PDO-derived cancer cells and cancer-associated fibroblasts (CAFs).

SUMMARY

The tumor microenvironment (TME) comprises all non-tumor elements of cancer and strongly influences disease progression and phenotype. To understand tumor biology and accurately test new therapeutic strategies, representative models should contain both tumor cells and normal cells of the TME. Here, we describe and characterize co-culture tumor-derived organoids and cancer-associated fibroblasts (CAFs), a major component of the TME, in matrix-embedded assembloid models of esophageal adenocarcinoma (EAC). We demonstrate that the assembloid models faithfully recapitulate the differentiation status of EAC and different CAF phenotypes found in the EAC patient TME. We evaluate cell phenotypes by combining tissue-clearing techniques with whole-mount immunofluorescence and histology, providing a practical framework for the characterization of cancer assembloids.

INTRODUCTION

Esophageal cancer is the sixth leading cause of cancer-related deaths worldwide, with more than 500,000 new cases reported annually.[1] Esophageal adenocarcinoma (EAC) is the dominant subtype in Western countries, and due to its late-stage presentation and therapy resistance, EAC is a deadly cancer with a 5-year survival of less than 15%. Risk factors include gastroesophageal reflux disease, Barrett's esophagus, obesity, and alcohol and tobacco consumption.[2] Surgery represents the primary treatment for early-stage disease, whereas patients with locally advanced disease are treated with perioperative chemotherapy[3] or chemoradiotherapy prior to surgery.[4] Despite rapid advances in molecularly targeted treatments in other cancers, progress in EAC has been slow, with only HER2-directed therapies entering clinical practice.[5] Most recently, immunotherapies, in the form of immune checkpoint inhibitors, have entered the field, but durable responses are rare.[6] The intra- and inter-tumoral heterogeneity of the disease presents an obstacle for introducing new therapies, which may partly be attributed to the lack of physical models that reflect the primary disease.

© 2024 The Author(s). Published by Elsevier Inc.
This is an open access article under the CC BY license (http://creativecommons.org/licenses/by/4.0/).

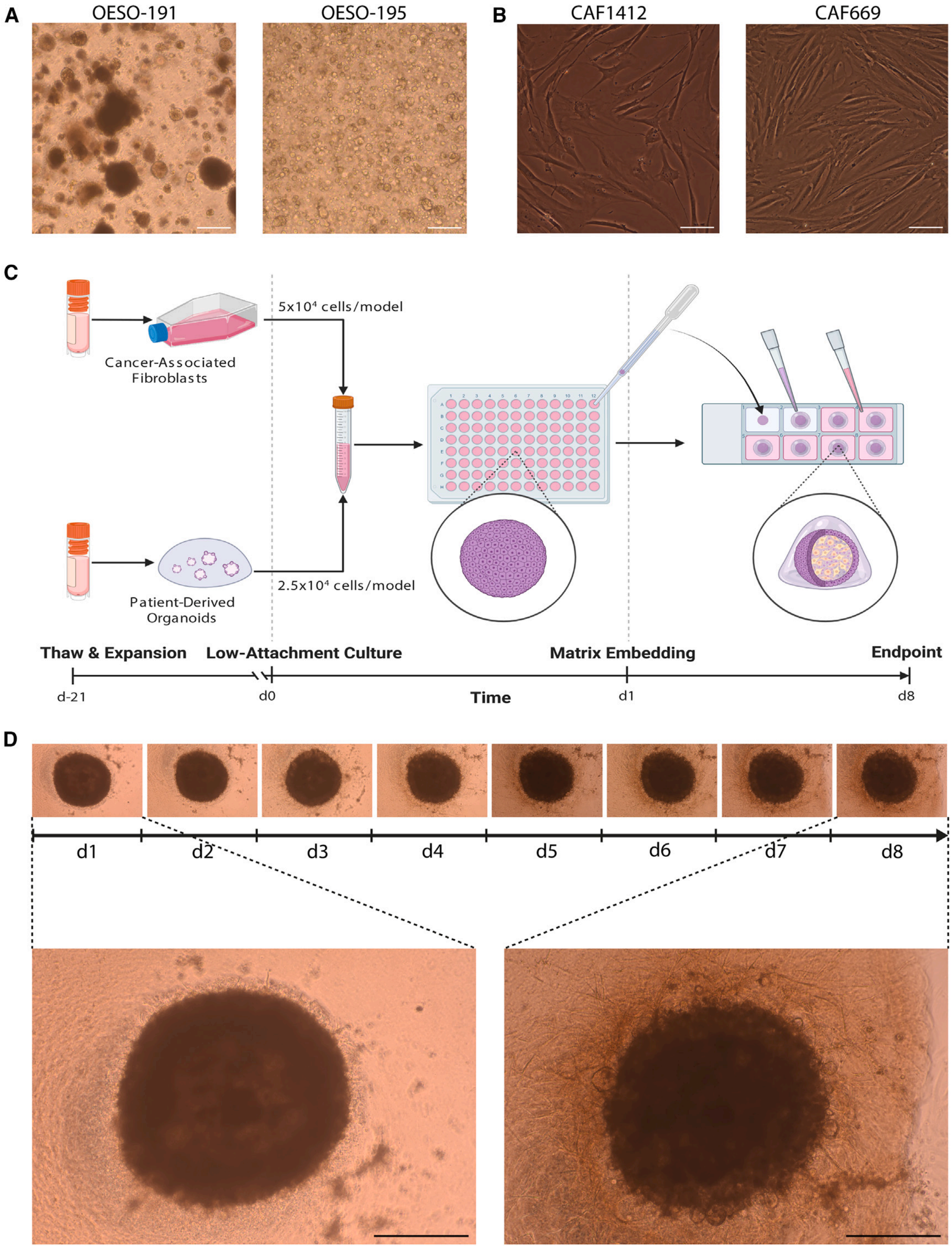

Figure 1. Derivation of primary models and the workflow for creation of EAC assembloids

(A) Two example EAC organoids derived from different patients with EAC observed with a 4× objective under phase contrast microscopy. Scale bars: 250 μm.

(B) Two example CAFs derived from different patients with EAC observed with a 20× objective under phase contrast microscopy. Scale bars: 50 μm.

(C) Workflow for production of EAC assembloids. EAC organoids and primary CAFs are expanded prior to model creation, dissociated to single cells, counted, and mixed together at a 2:1 ratio of CAFs to organoid cells. The cell suspension is then plated out at 75,000 cells per well in an ultra-low attachment 96-well

(legend continued on next page)

There is accumulating evidence that the tumor microenvironment (TME), which comprises components including immune cells, fibroblasts, endothelial cells, adipocytes, and extracellular matrix (ECM), contributes to tumorigenesis as well as therapy resistance.[7] Among these, cancer-associated fibroblasts (CAFs) make up a large proportion of the TME.[8] CAFs help control cancer progression, in addition to influencing cancer cell proliferation, ECM remodeling, metastasis, and therapy resistance, through the secretion of growth factors and ECM.[9] Clinically, CAF positivity in EAC was associated with worse tumor stage and a higher rate of metastasis in addition to shorter disease-free and overall survival.[10] We have previously shown that markers of myofibroblast CAF differentiation, α-smooth muscle actin (α-SMA), and periostin (POSTN) are associated with poor prognosis in EAC.[8] Targeting myofibroblast differentiation in EAC CAFs can be achieved in preclinical models using PDE5 inhibitors and sensitizes tumor cells to chemotherapy.[11] These studies highlight the importance of the EAC TME in governing tumor behavior, and the TME must be considered when studying drug sensitivity, which current EAC models are lacking.[12] Close-to-patient models that replicate the cancer-stroma interactions to understand the mechanism of resistance would be beneficial for the development of personalized treatment of EAC.

Patient-derived organoids (PDOs) have a wide range of applications in cancer research, including drug testing, personalized or precision medicine, and cancer immunotherapies. Cancer organoids are self-organizing cancer cells derived from patient tumors that recapitulate the structure, heterogeneity, histology, and genetic signatures of the primary tumor. PDOs have been successfully established in a variety of cancer types, including pancreatic,[13] colorectal,[14] and EAC.[15] The major limitation of these PDO models is the lack of TME components, which contribute to various hallmarks of cancer and response to therapy.[16,17] To recapitulate the cancer-stroma crosstalk, PDOs may be co-cultured with stromal cells, including CAFs.[18,19]

Studies in PDAC show that the co-culture of organoid models with pancreatic stellate cells (precursors of CAFs) resulted in the presence of myofibroblastic and inflammatory CAF populations,[20,21] phenotypes that are observed in patients with PDAC.[22] A study by Seino et al. revealed that CAFs could supply Wnt to support the growth of a Wnt-non-secreting subtype of PDAC PDOs.[23] The addition of CAFs also increased the proliferation and resistance of PDAC PDOs to chemotherapies.[19] In colorectal carcinoma (CRC), CAFs were able to maintain the proliferation of CRC PDO co-cultures and restored key survival pathways and cancer-CAF interactions present in the patient tissue.[24] Additionally, CRC PDO-CAF co-cultures show an enhanced resistance to standard-of-care drugs.[24] These data are evidence of the crosstalk between the organoids and CAFs and highlights the importance of incorporating CAFs when modeling tumor biology *in vitro*. While EAC PDO models have been established,[15] models that incorporate patient-derived CAFs are lacking. Organotypic culture systems, which consist of a collagen-fibroblast layer and epithelial cells plated over a collagen-rich matrix *in vitro*, may be used to model epithelia-stroma crosstalk in the esophagus.[8,25] Organotypics recapitulate the organization of a squamous epithelium and underlying stroma but require many cells for construction, and their large physical size limits imaging capabilities and restricts downstream assays to dissociated cells and histological sections. These challenges make it difficult to study the three-dimensional (3D) organization and crosstalk within organotypics, especially when using short-lived primary cells such as fibroblasts. Smaller models that are tractable for 3D imaging are required to study tumor-stroma interactions in 3D at cellular resolution.

In this study, we characterized EAC PDOs and devised a co-culture method using basement-membrane matrix and collagen to enable the co-culture of EAC PDOs with patient-derived CAFs to create self-organizing EAC assembloids.[26] We present an optimized method for non-destructive immunofluorescent staining and imaging of assembloids to facilitate the study of CAF-PDO interactions in 3D. We show that these assembloids are a viable approach to model cancer-stromal interactions *in vitro*.

RESULTS

Generation of EAC organoid-fibroblast assembloids

To study the interactions of the tumor epithelium with CAFs, we adapted a protocol established by Seino and colleagues to co-culture pancreatic cancer organoids with fibroblasts.[23,25] PDOs were derived from EAC tissue at resection following representative treatment pathways (neoadjuvant chemotherapy followed by esophagectomy). Fibroblasts were derived and expanded from EAC resection material to generate CAFs from EAC tissue via explant outgrowth as previously described.[25]

Grown in 3D culture with basement membrane extract (BME) and esophageal organoid growth media, EAC PDOs grow with diverse morphologies ranging from dark and dense to pale and cystic tumor buds (Figure 1A). Primary EAC CAFs grown in 2D display a classical elongated, spindle-like morphology (Figure 1B). The two cell types were co-cultured in a 2:1 ratio of CAFs:PDOs as established by Seino and colleagues,[25] starting with 2.5×10^4 organoid cells and 5×10^4 CAFs per model (Figure 1C). Co-cultures were grown in complete DMEM, avoiding the use of expensive esophageal organoid growth media, which contains factors that maintain epithelial stem cell niches[27] and therefore may affect the phenotype of CAFs in culture. Esophageal PDOs do not survive when grown in BME in complete DMEM in the absence of fibroblasts (data not shown), suggesting that CAFs provide factors essential for survival and proliferation of tumor cells *in vitro* as they would *in vivo*.[23] After overnight low-attachment culture conditions facilitating cell aggregation, co-cultures were embedded in a mix of 3:1 rat collagen I:BME2, fed with complete DMEM, and cultured for 7 further days *in vitro*. Co-cultures are compact and dense on

U-bottom plate. The next day, assembloids are plated in a 3:1 mixture of collagen I:BME2, and complete DMEM media are overlayed once set. Assembloids are cultured for a further 7 days prior to harvest.

(D) Structure formation of EAC assembloids imaged with a 4× objective under phase contrast microscopy every day for 7 days after matrix embedding. Scale bars: 500 μm.

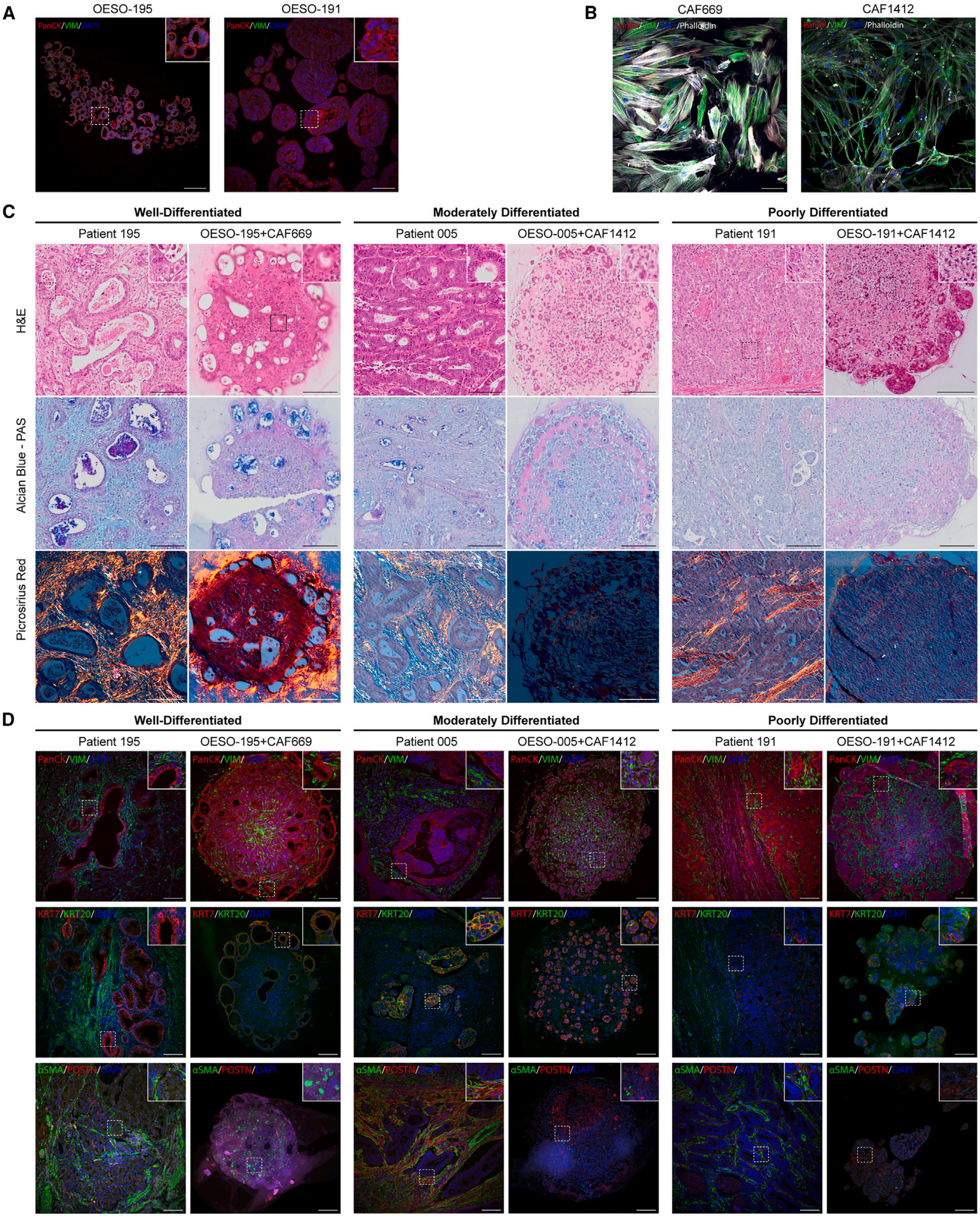

(legend on next page)

day 1, initially contracting and then developing round bud-like structures on the periphery of cultures on day 3, which continue to develop until endpoint analysis at day 8 (Figure 1D). Fibroblasts grow out of the co-culture, and by day 8, they also occupy the surrounding gel.

PDO-CAF assembloids recapitulate features of primary tumors

To verify the epithelial or mesenchymal nature of the input primary tumor cells, we embedded organoids in agarose and subjected them to formalin-fixed, paraffin-embedded (FFPE) tissue processing and immunofluorescence (IF) to detect epithelial cytokeratin (using pan-cytokeratin antibody clone AE1/AE3) and the mesenchymal marker vimentin.[28] Similarly, fibroblasts were seeded onto 8-well chamber slides and stained for pan-cytokeratin and vimentin. EAC organoids were heterogeneously positive for epithelial cytokeratins but negative for vimentin (Figure 2A), and fibroblasts stained conversely (Figure 2B), consistent with their distinct epithelial and stromal origins.

We grew three different assembloids containing combinations of three different organoids and two different CAFs for 8 days and processed these for conventional FFPE histology alongside FFPE parental tumor material from esophagectomy. We performed a panel of tinctorial stains to assess the histological composition of the assembloids compared to the parent tumor: H&E staining to assess histological differentiation, Alcian blue and periodic acid Schiff (AB-PAS) for the detection of mucins commonly secreted by EAC tumors, and picrosirius red (PSR) to detect collagen fiber deposition and alignment under polarized light (Figure 2C).

Histopathological evaluation of H&E stains revealed similar histological differentiation between the assembloids and parent tumors, with additional differences in histology of assembloids between central and peripheral regions (Figure 2C). Patient 195 had a well-differentiated adenocarcinoma with large hollow tumor acini with intraluminal accumulation of acidic (stained blue) and neutral (stained purple) mucins. Assembloids of organoid OESO-195 with CAFs produced similar phenotypes under H&E and AB-PAS stains, with accumulation of tumor acini on the peripheral regions and accumulation of necro-inflammatory debris in the central region of the assembloid. This is suggestive of central hypoxia and an organization of proliferating cells on the outer layers, while cell death occurs centrally. Collagen deposits stained with PSR (orange birefringence) were visible surrounding the outer layers of assembloids containing OESO-195 and CAF669 but were not visible in assembloids containing CAF1412 (Figure 2C). Unlike parent tumors, no assembloids displayed mature collagen networks with aligned collagen fibers, suggesting an absence of collagen-secreting fibroblast phenotypes in these assembloids.

Patient 005 had a well- to moderately differentiated adenocarcinoma containing dense tumor acini and smaller lumens with acidic mucin accumulation under AB-PAS stain (Figure 2C). Assembloids of organoid OESO-005 displayed moderate differentiation with smaller acinar structures than the parent tumor and acidic-mucin-containing lumens distributed throughout the culture. The central region of the assembloid also contained singly dispersed tumor cells with a more poorly differentiated morphology than the peripheral region. Although the corresponding patient tumor forms much larger acini, the overall differentiation status is equivalent to the assembloid, being moderately to well differentiated.

Patient 191 had a poorly differentiated adenocarcinoma with disorganized tumor nests, notably absent lumens, and no mucin secretion detectable by AB-PAS (Figure 2C). Similarly, an assembloid culture of organoid OESO-191 displayed poor differentiation with solid tumor nests in the peripheral region and singly dispersed cells with signet ring cell-like morphology in the central region (Figure 2C, arrowheads). A signet ring cell-like morphology indicates intracellular accumulation of mucins, which push aside the nucleus, and usually indicates poor clinical behavior. Interestingly, sections derived from the parent tumor did not have observable signet ring histology, which may suggest an additional effect of assembloid culture conditions on phenotypic heterogeneity at the histological level.

To ensure that the cancer and fibroblast cell types present in the assembloid were representative of patients, we next performed duplex IF on the same FFPE blocks from parent tumor and matched assembloids (Figure 2D, insets). Staining for pan-cytokeratin and vimentin suggested that vimentin+ fibroblasts surrounded cytokeratin+ tumor acini in assembloids as they do in the parent tumor. The central region was particularly fibroblast dense, and cytokeratin+ tumor acini preferentially localized to the periphery, especially in the well-differentiated OESO-195 assembloid. EAC tumor cells are thought to be cytokeratin 7 (CK7)+ but are usually negative for CK20.[29] Assembloids mirrored the cytokeratin expression patterns of their parent tumors (Figure 2D, insets). Patient 195 was CK7+/CK20−, and the corresponding assembloid reflected this; patient 005 was heterogeneously positive for both CK7 and CK20, whereas the assembloid was mostly CK7+/CK20−, with a minority of CK7+/CK20+ tumor cells. Patient 191 had lower levels of CK7 expression, and the

Figure 2. Histological and immunohistochemical characterization of EAC assembloid phenotypes compared to the corresponding patient tumors

(A) Immunohistochemistry of 5 μm organoid sections grown in monoculture and stained for pan-cytokeratin (red) and vimentin (green). Nuclei were counterstained with DAPI (blue).

(B) Immunocytochemistry of primary fibroblasts grown in monoculture and stained for pan-cytokeratin (red) and vimentin (green). Actin cytoskeleton is counterstained with phalloidin (grayscale), and nuclei are counterstained with DAPI (blue). Scale bars: 100 μm.

(C) 5 μm sections of EAC assembloids and resected patient tumors stained with tinctorial stains for overall histology (H&E), mucin secretion (Alcian blue-PAS, mucins in blue/purple), and collagen fibers (picrosirius red, collagen in orange) viewed under polarized light. Signet ring-like cells are indicated in the inset of OESO-191+CAF1412 with arrowheads.

(D) Immunofluorescence of EAC assembloids and resected patient tumor sections stained for markers of epithelial and fibroblast cell identity—pan-cytokeratin and vimentin, keratin 7 (glandular epithelium) and keratin 20 (colonic epithelium), and α-SMA and POSTN (myofibroblast differentiation). Nuclei are counterstained with DAPI. Insets show regions of interest at higher resolution in (A), (C), and (D). Scale bars: 150 μm.

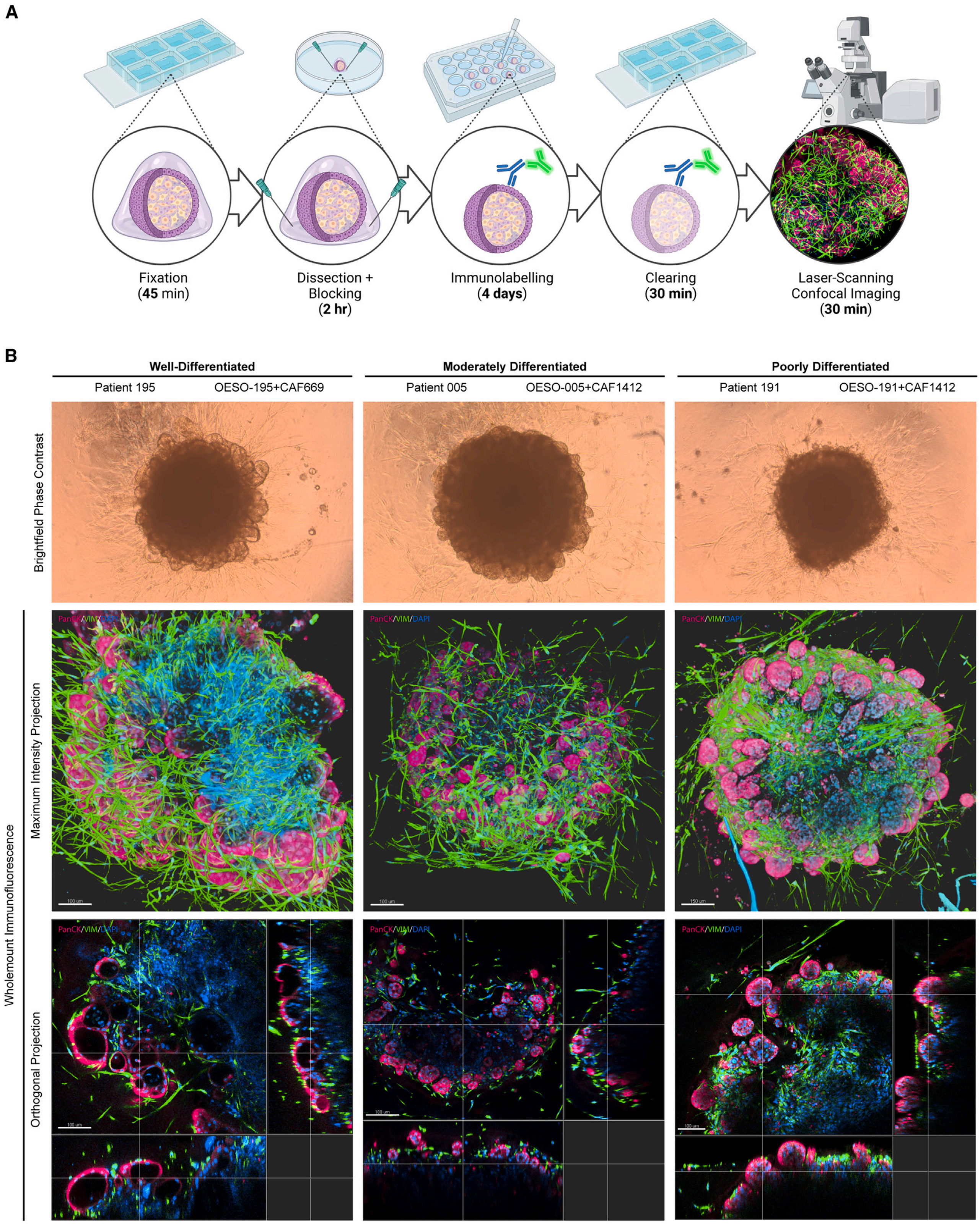

(legend on next page)

OESO-191 assembloid contained an admixture of CK7+ and CK20+ tumor cells. Next, we examined the expression of myofibroblast CAF markers α-SMA and POSTN, as these are known to be associated with poor prognosis in EAC.[8,11] Assembloids containing OESO-005 and OESO-191 grown with CAF1412 produced an SMA− and POSTN-low stromal microenvironment (Figure 2D, insets), whereas the stromal microenvironment of OESO-195 grown with CAF669 produced a stromal microenvironment with SMA+ cells distributed throughout, suggesting some myofibroblast CAF differentiation in this model. To characterize our assembloids and their respective components, we performed bulk RNA sequencing (RNA-seq) on the two CAFs, three organoid lines, and corresponding assembloid co-cultures for one CAF (CAF669) with all three organoids (Figures S1 and S2A). Principal-component analysis reveals that CAF and organoid transcriptomes are more similar to other CAFs and organoids, respectively, whereas assembloid transcriptomes occupy a distinct space, with OESO-005 assembloids more strongly influenced by co-culture than the other two lines (Figure S1B). We clustered gene expression profiles to observe patterns of expression induced by assembloid culture. Assembloid gene expression profiles contained unique clusters (clusters 1, 2, and 6; Figure S1B; Table S1) distinct from parental CAFs and organoids. Bulk RNA-seq from the CAFs indicated that CAF669 may be more inflammatory than CAF1412 (Figure S2A). We therefore performed RT-qPCR on the two CAFs with a panel of CAF phenotype markers known to be present in patients with EAC.[30] However, both CAFs display expression of α-SMA, indicating that both CAFs represent a more myofibroblastic CAF-like phenotype (Figure S2B). Finally, to identify whether assembloids could potentially be used to study hypoxia in the context of EAC, we analyzed the RNA-seq data for expression of hypoxia markers compared to organoids alone (Figure S3A). The data indicate that assembloids express significantly higher levels of hypoxia markers compared to organoids alone (Figure S3B, $p < 0.01$).

Whole-mount IF demonstrates organization of fibroblasts and morphological variation between patient co-cultures

3D models provide the most information where 3D context and gene/protein expression can be related. However, thick specimens limit the diffusion of staining reagents and the penetration of light, in part due to the presence of lipids.[31] We developed a non-destructive protocol for whole-mount IF that permits the penetration of antibodies and visualization up to ~250 μm depth into the assembloid models (Figure 3A). We adapted this method from Dekkers et al.,[32] using FLASH (fast light-microscopic analysis of antibody-stained whole organs) antigen retrieval from Messal et al.[33] to improve antibody penetration and imaging depth for large models. The staining protocol takes 5 days, and stained specimens can be stored in clearing buffer at 4°C for >2 weeks prior to imaging. Briefly, excess collagen gel was dissected from the assembloids using needles, and we performed antigen retrieval using FLASH reagent 2[33] for 2 h at 37°C. This is an SDS and zwitterionic detergent-based method to remove tissue lipids and improve the reagent and light penetration through the specimen. To indirectly immunolabel co-cultures, we incubated with primary antibodies for 2 days at 4°C and then for a further 2 days with secondary antibodies and DAPI for nuclear counterstaining. To clear the assembloids for imaging, we immersed them for at least 30 min in fructose-glycerol clearing buffer[32] (60% [v/v] glycerol and 2.5 M fructose, refractive index = 1.47) and then imaged them with a laser scanning confocal microscope. To demonstrate the utility of this method, we whole-mount-stained assembloids with anti-pan-cytokeratin and anti-vimentin to visualize both compartments in a native 3D context. Budding tumor structures visible by bright-field observation are revealed in detail when imaged by whole-mount IF, showing discrete pan-cytokeratin+ structures on the periphery of assembloids (Figure 3B; Videos S1, S2, and S3), with internal lumens in the well- and moderately differentiated assembloids and solid tumor masses in poorly differentiated co-cultures. Vimentin+ fibroblasts can be seen in close association with tumor acini, and areas of denser fibroblast presence are more apparent in maximum-intensity projections of whole-mount-IF-stained samples than in paraffin sections. The proliferation of cancer cells within assembloids became apparent when whole-mount staining with anti-Ki-67, with poorly differentiated assembloids having the greatest abundance of proliferating cells (Figure 4).

DISCUSSION

Organoid cultures preserve the heterogeneity and molecular characteristics of the primary tumor and facilitate testing treatment strategies and understanding responses to them in EAC.[15] However, current models do not incorporate stromal elements of the TME, which provide context cues by both paracrine and juxtacrine interactions. Furthermore, it is well recognized that stromal cells, including CAFs, play a major role in tumor progression and drug response.[34] Assembloids are an emerging technology for the study of epithelial cells within their mesenchymal niches, with assembloid models recently being developed for modeling stromal interactions in the murine normal and malignant colon.[35] Our model combines the versatility of patient-derived tumor organoid lines with

Figure 3. Whole-mount immunofluorescent protocol for 3D visualization of EAC assembloid organization
(A) Workflow of the 5-day whole-mount staining protocol. EAC assembloids are fixed in 4% PFA, excess collagen is removed to improve antibody penetration, and then assembloids are stained with primary and fluorescent-conjugated secondary antibodies (with DAPI) for 2 days each. Assembloids are cleared in fructose-glycerol clearing buffer and then imaged with a fluorescent laser scanning confocal microscope.
(B) Example assembloids from each organoid/CAF combination at day 8 of culture imaged in bright-field phase contrast (scale bars: 500 μm). Shown below are corresponding Z-projections of whole-mount-stained and cleared assembloids for pan-cytokeratin (organoid cells, red) and vimentin (fibroblasts, green) in top-down maximum intensity projections and orthogonal projections cutting through outer epithelial buds (scale bars: 100 μm unless indicated otherwise). Rendering was performed in Imaris under blend mode.
See also Videos S1, S2, and S3.

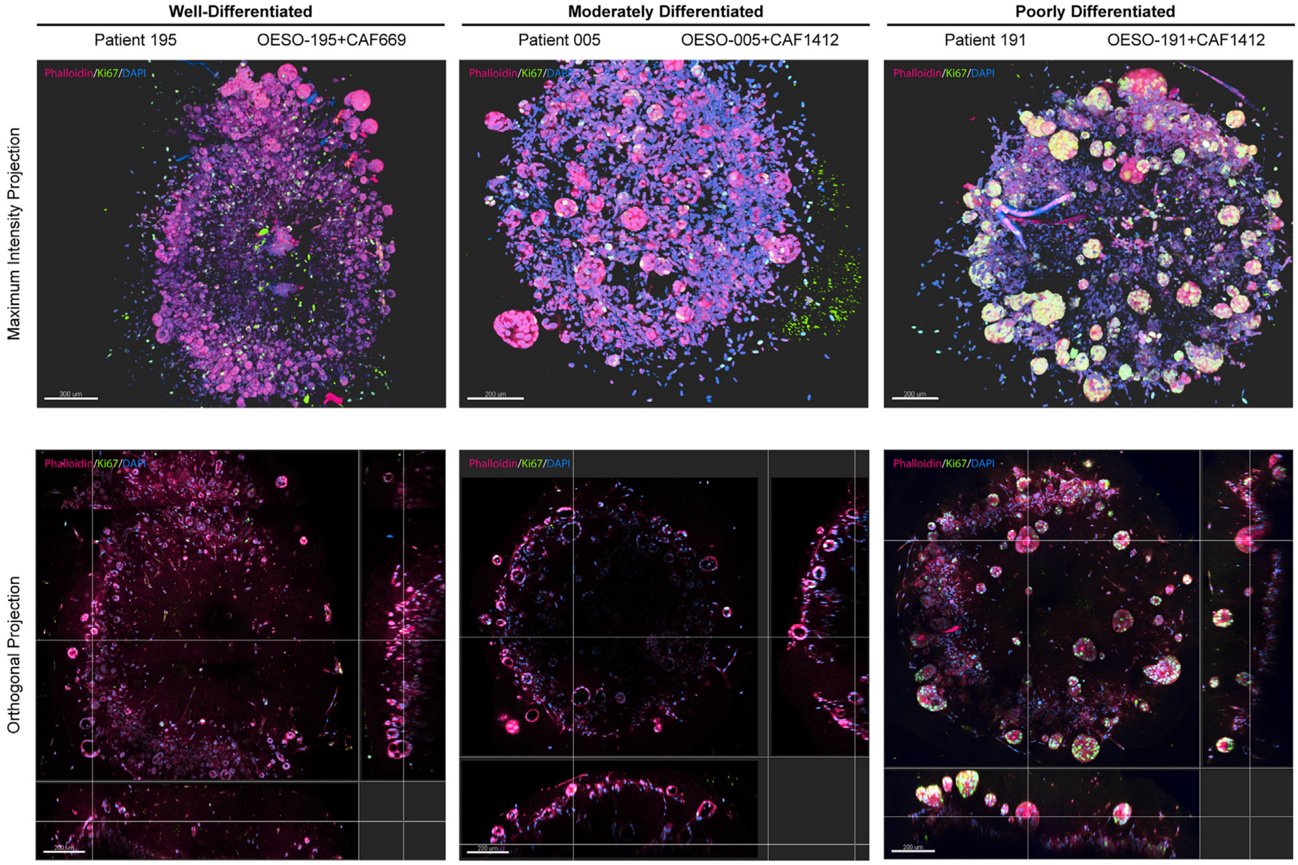

Figure 4. Visualization of cell proliferation in 3D using whole-mount immunofluorescence

Z-projections of whole-mount-stained and cleared 8-day-old assembloids for Ki-67 (proliferating cells, green) counterstained with Phalloidin-iFluor594 (cell bodies, red) in top-down maximum intensity projections and orthogonal projections cutting through outer epithelial buds (scale bars: 200 μm). Rendering was performed in Imaris under blend mode.

well-characterized genomic and transcriptional landscapes with primary CAFs from heterologous donors. This method facilitates physical contact of CAFs with tumor cells where juxtacrine signaling can take place, as opposed to other methods where fibroblasts loosely associate due to being mixed into the ECM.[36] The models are low to medium throughput, technically simple to produce, require no specialized equipment, and would accommodate the addition of other cell types to suit the research question (e.g., immune or vascular cells).

We show that EAC assembloids recapitulated features of the primary tumor, including histology and differentiation status, while recapitulating tumor-stroma interactions. We observed that CAFs support the survival and proliferation of EAC PDOs in complete DMEM, crucially enabling low-cost model production and scalability. This was also observed in pancreatic ductal adenocarcinoma (PDAC),[23] normal colon,[35] and CRC,[24] where growth of the epithelial compartment was supplemented by fibroblasts even in the absence of exogenous Wnt and BMP signaling activators. We observed spatial arrangements of EAC assembloid models comparable to those seen in multicellular tumor spheroids, where reduced access to oxygen and nutrients internally causes models to proliferate in peripheral areas, with central quiescence and necrosis as spheroids grow larger.[37] Progressive growth of tumor buds from the periphery of EAC assembloids and central necro-inflammatory debris suggested a similar dynamic occurring in these models. Interestingly, histology revealed the presence of singly dispersed tumor cells and signet ring cells in central zones of EAC assembloids, which are features associated with poor prognosis in tumors.[38] These did not match with patient tumor histology, and given the large size of the models, we suggest that they might be generated by hypoxia within the dense fibroblast-rich core. Hypoxia has previously been reported to push CAFs toward an inflammatory phenotype when co-cultured with mouse PDAC organoids while leaving myofibroblast markers unaffected.[39] Simulating a hypoxic environment using assembloids offers the opportunity to better understand the impact of hypoxia on fibroblast and organoid phenotypes and the crosstalk between them.

In this study, we successfully established a disease relevant co-culture model consisting of patient derived CAFs and organoids that enables technically straightforward modeling of tumor-stromal crosstalk. We combined the versatility of patient-derived tumor organoid lines with well-characterized genomic and transcriptional landscapes with primary CAFs from

CellPress
OPEN ACCESS

Table 1. Clinical information for each patient from whom primary fibroblasts or organoids were derived and references to associated cell culture and genomic information

Patient	Organoid	Sanger Cell Model Passports ID	CAF	Age	Sex	Histology	Clinical stage	Pathological stage	Neoadjuvant treatment	Tumor regression grade
005	OESO-005	HCM-SANG-0290-C15	N/A	68	M	adenocarcinoma	cT3N2M0	ypT3N2M0	ECX	4
191	OESO-191	HCM-SANG-0543-C15	N/A	60	M	adenocarcinoma	cT3N0M0	ypT3N0M0	CAPOX	4
195	OESO-195	HCM-SANG-0546-C15	N/A	72	M	adenocarcinoma	cT2N1M0	ypT2N1M0	FLOT	3
669	N/A	N/A	CAF669	71	F	adenocarcinoma	cT2N0M0	pT2N0M0	none	N/A
1412	N/A	N/A	CAF1412	81	M	adenocarcinoma	cT3N1M0	pT3N3M0	none	N/A

M, male; F, female; ECX, epirubicin, cisplatin, and capecitabine; CAPOX, capecitabine and oxaliplatin; FLOT, 5-fluorouracil, leucovorin, oxaliplatin, and docetaxel.

heterologous donors. We developed a non-destructive and inexpensive clearing and imaging toolkit that enables the visualization of interactions in co-cultures in 3D.

Limitations of the study

CAFs are recognized to be heterogeneous with different subtypes and different functions in the TME intratumorally.[40] Our study is limited due to the CAFs being heterologous, therefore not fully mimicking CAF-PDO interactions, as they are present in the patient from which the organoid was derived. However, we have shown that CAFs maintain phenotypes observed in monoculture when grown in assembloids. This provides the opportunity to study the contribution of diverse CAF phenotypes from different patient subgroups to tumor cell behavior. Future work aims to use matched EAC PDOs and CAFs to develop patient-specific models as a prediction of drug response, which will be correlated to clinical outcomes, as well as explore the incorporation of matched immune cells to develop a co-culture model for testing immunotherapies. Furthermore, the current method is low throughput, being able to generate <96 models in one experiment and limited by manual handling. To facilitate higher-throughput screening efforts, which include stromal populations, miniaturization and reducing manual transfer steps in matrix embedding would be required.

RESOURCE AVAILABILITY

Lead contact

Further information and requests for resources and reagents should be directed to the lead contact, Tim J. Underwood (t.j.underwood@soton.ac.uk).

Materials availability

All reagents generated in this study will be made available by the lead contact with an appropriate materials transfer agreement (MTA).

Data and code availability

- Raw and processed bulk RNA-seq data have been deposited at GEO and are publicly available as of the date of publication with accession number GEO: GSE277147. Microscopy data have been deposited at Zenodo, and DOIs are listed in the key resources table.
- This paper does not report original code.
- Any additional information required to reanalyze the data reported in this work is available from the lead contact upon request.

ACKNOWLEDGMENTS

The authors thank patients at the University Hospital Southampton for their participation in this study, Dr. Mathew Garnett and the Cellular Generation and Phenotyping laboratory at Wellcome Trust Sanger Institute for organoid derivation, and the Faculty of Medicine Tissue Bank at the University of Southampton for patient material and organoid biobanking, transport, and tracking. FFPE tissue blocks were curated by Research Histology, Department of Cell Pathology, University Hospital Southampton. Histology was supported by the Histochemistry Research Unit, University of Southampton. Bioimaging, including slide scanning and laser scanning confocal microscopy, was supported by the Biomedical Imaging Unit, University Hospital Southampton. The authors gratefully acknowledge the Faculty of Medicine BIO-R at the University of Southampton for their support and assistance with bioinformatic analyses. T.J.U. is supported by a Royal College of Surgeons of England and Cancer Research UK Advanced Clinician Scientist Fellowship (A23924). Schematics in the figures were created with BioRender.com.

AUTHOR CONTRIBUTIONS

Conceptualization, B.P.S., Z.S.W., and T.J.U.; methodology, B.P.S. and D.A.J.; formal analysis, J.T.; investigation, B.P.S., L.A.N., C.T., D.A.J., R.B., J.H., R.R., and M.R.-Z.; resources, D.A.J. and T.J.U.; data curation, O.J.P. and C.T.; writing – original draft, B.P.S., C.T., and Z.S.W.; writing – review & editing, B.P.S., C.T., M.R.-Z., Z.S.W., and T.J.U.; visualization, B.P.S., D.A.J., J.T., and M.R.Z.; supervision, T.J.U. and Z.S.W.; project administration, B.P.S., Z.S.W., and T.J.U.; funding acquisition, T.J.U.

DECLARATION OF INTERESTS

The authors declare no competing interests.

STAR★METHODS

Detailed methods are provided in the online version of this paper and include the following:

- KEY RESOURCES TABLE
- EXPERIMENTAL MODEL AND STUDY PARTICIPANT DETAILS
  - Patient cohort
  - Cell lines
- METHOD DETAILS
  - Derivation of patient-derived organoids from EAC tissue
  - Derivation of primary fibroblasts from EAC tissue
  - Production of assembloid models
  - Wholemount immunofluorescence on assembloid models

- ○ Agarose embedding of organoids and assembloids for paraffin histology
- ○ Hematoxylin & eosin staining
- ○ Alcian blue – Periodic acid schiff staining
- ○ Picro-sirius red staining
- ○ Double immunofluorescence on FFPE embedded tissue and organoids
- ○ qRT-PCR of CAFs
- ○ Bulk RNA-sequencing of organoids, CAFs and assembloids
- QUANTIFICATION AND STATISTICAL ANALYSIS

SUPPLEMENTAL INFORMATION

Supplemental information can be found online at https://doi.org/10.1016/j.crmeth.2024.100909.

Received: January 4, 2024
Revised: July 1, 2024
Accepted: November 6, 2024
Published: November 27, 2024

REFERENCES

1. Sung, H., Ferlay, J., Siegel, R.L., Laversanne, M., Soerjomataram, I., Jemal, A., and Bray, F. (2021). Global Cancer Statistics 2020: GLOBOCAN Estimates of Incidence and Mortality Worldwide for 36 Cancers in 185 Countries. CA A Cancer J. Clin. *71*, 209–249. https://doi.org/10.3322/caac.21660.
2. Uhlenhopp, D.J., Then, E.O., Sunkara, T., and Gaduputi, V. (2020). Epidemiology of esophageal cancer: update in global trends, etiology and risk factors. Clin. J. Gastroenterol. *13*, 1010–1021. https://doi.org/10.1007/s12328-020-01237-x.
3. Al-Batran, S.E., Homann, N., Pauligk, C., Goetze, T.O., Meiler, J., Kasper, S., Kopp, H.G., Mayer, F., Haag, G.M., Luley, K., et al. (2019). Perioperative chemotherapy with fluorouracil plus leucovorin, oxaliplatin, and docetaxel versus fluorouracil or capecitabine plus cisplatin and epirubicin for locally advanced, resectable gastric or gastro-oesophageal junction adenocarcinoma (FLOT4): a randomised, phase 2/3 trial. Lancet *393*, 1948–1957. https://doi.org/10.1016/s0140-6736(18)32557-1.
4. Eyck, B.M., van Lanschot, J.J.B., Hulshof, M.C.C.M., van der Wilk, B.J., Shapiro, J., van Hagen, P., van Berge Henegouwen, M.I., Wijnhoven, B.P.L., van Laarhoven, H.W.M., Nieuwenhuijzen, G.A.P., et al. (2021). Ten-Year Outcome of Neoadjuvant Chemoradiotherapy Plus Surgery for Esophageal Cancer: The Randomized Controlled CROSS Trial. J. Clin. Oncol. *39*, 1995–2004. https://doi.org/10.1200/jco.20.03614.
5. Bang, Y.J., Van Cutsem, E., Feyereislova, A., Chung, H.C., Shen, L., Sawaki, A., Lordick, F., Ohtsu, A., Omuro, Y., Satoh, T., et al. (2010). Trastuzumab in combination with chemotherapy versus chemotherapy alone for treatment of HER2-positive advanced gastric or gastro-oesophageal junction cancer (ToGA): a phase 3, open-label, randomised controlled trial. Lancet *376*, 687–697. https://doi.org/10.1016/s0140-6736(10)61121-x.
6. Janjigian, Y.Y., Shitara, K., Moehler, M., Garrido, M., Salman, P., Shen, L., Wyrwicz, L., Yamaguchi, K., Skoczylas, T., Campos Bragagnoli, A., et al. (2021). First-line nivolumab plus chemotherapy versus chemotherapy alone for advanced gastric, gastro-oesophageal junction, and oesophageal adenocarcinoma (CheckMate 649): a randomised, open-label, phase 3 trial. Lancet *398*, 27–40. https://doi.org/10.1016/s0140-6736(21)00797-2.
7. Schiffmann, L.M., Plum, P.S., Fuchs, H.F., Babic, B., Bruns, C.J., and Schmidt, T. (2021). Tumor Microenvironment of Esophageal Cancer. Cancers *13*, 4678. https://doi.org/10.3390/cancers13184678.
8. Underwood, T.J., Hayden, A.L., Derouet, M., Garcia, E., Noble, F., White, M.J., Thirdborough, S., Mead, A., Clemons, N., Mellone, M., et al. (2015). Cancer-associated fibroblasts predict poor outcome and promote periostin-dependent invasion in oesophageal adenocarcinoma. J. Pathol. *235*, 466–477. https://doi.org/10.1002/path.4467.
9. Sahai, E., Astsaturov, I., Cukierman, E., DeNardo, D.G., Egeblad, M., Evans, R.M., Fearon, D., Greten, F.R., Hingorani, S.R., Hunter, T., et al. (2020). A framework for advancing our understanding of cancer-associated fibroblasts. Nat. Rev. Cancer *20*, 174–186. https://doi.org/10.1038/s41568-019-0238-1.
10. Schoppmann, S.F., Jesch, B., Riegler, M.F., Maroske, F., Schwameis, K., Jomrich, G., and Birner, P. (2013). Podoplanin expressing cancer associated fibroblasts are associated with unfavourable prognosis in adenocarcinoma of the esophagus. Clin. Exp. Metastasis *30*, 441–446. https://doi.org/10.1007/s10585-012-9549-2.
11. Sharpe, B.P., Hayden, A., Manousopoulou, A., Cowie, A., Walker, R.C., Harrington, J., Izadi, F., Breininger, S.P., Gibson, J., Pickering, O., et al. (2022). Phosphodiesterase type 5 inhibitors enhance chemotherapy in preclinical models of esophageal adenocarcinoma by targeting cancer-associated fibroblasts. Cell Rep. Med. *3*, 100541. https://doi.org/10.1016/j.xcrm.2022.100541.
12. Saunders, J.H., Onion, D., Collier, P., Dorrington, M.S., Argent, R.H., Clarke, P.A., Reece-Smith, A.M., Parsons, S.L., and Grabowska, A.M. (2017). Individual patient oesophageal cancer 3D models for tailored treatment. Oncotarget *8*, 24224–24236. https://doi.org/10.18632/oncotarget.12500.
13. Boj, S.F., Hwang, C.I., Baker, L.A., Chio, I.I.C., Engle, D.D., Corbo, V., Jager, M., Ponz-Sarvise, M., Tiriac, H., Spector, M.S., et al. (2015). Organoid models of human and mouse ductal pancreatic cancer. Cell *160*, 324–338. https://doi.org/10.1016/j.cell.2014.12.021.
14. van de Wetering, M., Francies, H.E., Francis, J.M., Bounova, G., Iorio, F., Pronk, A., van Houdt, W., van Gorp, J., Taylor-Weiner, A., Kester, L., et al. (2015). Prospective derivation of a living organoid biobank of colorectal cancer patients. Cell *161*, 933–945. https://doi.org/10.1016/j.cell.2015.03.053.
15. Li, X., Francies, H.E., Secrier, M., Perner, J., Miremadi, A., Galeano-Dalmau, N., Barendt, W.J., Letchford, L., Leyden, G.M., Goffin, E.K., et al. (2018). Organoid cultures recapitulate esophageal adenocarcinoma heterogeneity providing a model for clonality studies and precision therapeutics. Nat. Commun. *9*, 2983. https://doi.org/10.1038/s41467-018-05190-9.
16. Liu, J., Li, P., Wang, L., Li, M., Ge, Z., Noordam, L., Lieshout, R., Verstegen, M.M.A., Ma, B., Su, J., et al. (2021). Cancer-Associated Fibroblasts Provide a Stromal Niche for Liver Cancer Organoids That Confers Trophic Effects and Therapy Resistance. Cell. Mol. Gastroenterol. Hepatol. *11*, 407–431. https://doi.org/10.1016/j.jcmgh.2020.09.003.
17. Neal, J.T., Li, X., Zhu, J., Giangarra, V., Grzeskowiak, C.L., Ju, J., Liu, I.H., Chiou, S.H., Salahudeen, A.A., Smith, A.R., et al. (2018). Organoid Modeling of the Tumor Immune Microenvironment. Cell *175*, 1972–1988.e16. https://doi.org/10.1016/j.cell.2018.11.021.
18. Farin, H.F., Mosa, M.H., Ndreshkjana, B., Grebbin, B.M., Ritter, B., Menche, C., Kennel, K.B., Ziegler, P.K., Szabó, L., Bollrath, J., et al. (2023). Colorectal Cancer Organoid-Stroma Biobank Allows Subtype-Specific Assessment of Individualized Therapy Responses. Cancer Discov. *13*, 2192–2211. https://doi.org/10.1158/2159-8290.Cd-23-0050.
19. Schuth, S., Le Blanc, S., Krieger, T.G., Jabs, J., Schenk, M., Giese, N.A., Büchler, M.W., Eils, R., Conrad, C., and Strobel, O. (2022). Patient-specific modeling of stroma-mediated chemoresistance of pancreatic cancer using a three-dimensional organoid-fibroblast co-culture system. J. Exp. Clin. Cancer Res. *41*, 312. https://doi.org/10.1186/s13046-022-02519-7.
20. Biffi, G., Oni, T.E., Spielman, B., Hao, Y., Elyada, E., Park, Y., Preall, J., and Tuveson, D.A. (2019). IL1-Induced JAK/STAT Signaling Is Antagonized by TGFβ to Shape CAF Heterogeneity in Pancreatic Ductal Adenocarcinoma. Cancer Discov. *9*, 282–301. https://doi.org/10.1158/2159-8290.Cd-18-0710.
21. Öhlund, D., Handly-Santana, A., Biffi, G., Elyada, E., Almeida, A.S., Ponz-Sarvise, M., Corbo, V., Oni, T.E., Hearn, S.A., Lee, E.J., et al. (2017).

CellPress
OPEN ACCESS

Distinct populations of inflammatory fibroblasts and myofibroblasts in pancreatic cancer. J. Exp. Med. *214*, 579–596. https://doi.org/10.1084/jem.20162024.

22. Bernard, V., Semaan, A., Huang, J., San Lucas, F.A., Mulu, F.C., Stephens, B.M., Guerrero, P.A., Huang, Y., Zhao, J., Kamyabi, N., et al. (2019). Single-Cell Transcriptomics of Pancreatic Cancer Precursors Demonstrates Epithelial and Microenvironmental Heterogeneity as an Early Event in Neoplastic Progression. Clin. Cancer Res. *25*, 2194–2205. https://doi.org/10.1158/1078-0432.Ccr-18-1955.
23. Seino, T., Kawasaki, S., Shimokawa, M., Tamagawa, H., Toshimitsu, K., Fujii, M., Ohta, Y., Matano, M., Nanki, K., Kawasaki, K., et al. (2018). Human Pancreatic Tumor Organoids Reveal Loss of Stem Cell Niche Factor Dependence during Disease Progression. Cell Stem Cell *22*, 454–467.e6. https://doi.org/10.1016/j.stem.2017.12.009.
24. Luo, X., Fong, E.L.S., Zhu, C., Lin, Q.X.X., Xiong, M., Li, A., Li, T., Benoukraf, T., Yu, H., and Liu, S. (2021). Hydrogel-based colorectal cancer organoid co-culture models. Acta Biomater. *132*, 461–472. https://doi.org/10.1016/j.actbio.2020.12.037.
25. Underwood, T.J., Derouet, M.F., White, M.J., Noble, F., Moutasim, K.A., Smith, E., Drew, P.A., Thomas, G.J., Primrose, J.N., and Blaydes, J.P. (2010). A comparison of primary oesophageal squamous epithelial cells with HET-1A in organotypic culture. Biol. Cell. *102*, 635–644. https://doi.org/10.1042/bc20100071.
26. Vogt, N. (2021). Assembloids. Nat. Methods *18*, 27. https://doi.org/10.1038/s41592-020-01026-x.
27. Sato, T., Stange, D.E., Ferrante, M., Vries, R.G.J., Van Es, J.H., Van den Brink, S., Van Houdt, W.J., Pronk, A., Van Gorp, J., Siersema, P.D., and Clevers, H. (2011). Long-term expansion of epithelial organoids from human colon, adenoma, adenocarcinoma, and Barrett's epithelium. Gastroenterology *141*, 1762–1772. https://doi.org/10.1053/j.gastro.2011.07.050.
28. Sharma, P., Alsharif, S., Fallatah, A., and Chung, B.M. (2019). Intermediate Filaments as Effectors of Cancer Development and Metastasis: A Focus on Keratins, Vimentin, and Nestin. Cells *8*, 497. https://doi.org/10.3390/cells8050497.
29. Driessen, A., Nafteux, P., Lerut, T., Van Raemdonck, D., De Leyn, P., Filez, L., Penninckx, F., Geboes, K., and Ectors, N. (2004). Identical cytokeratin expression pattern CK7+/CK20- in esophageal and cardiac cancer: etiopathological and clinical implications. Mod. Pathol. *17*, 49–55. https://doi.org/10.1038/modpathol.3800011.
30. Walker, R., Breininger, S., Sharpe, B., Harrington, J., Reddin, I., Tse, C., Rajak, R., Hayden, A., Rahman, S., Grace, B., et al. (2023). Targetable fibroblast phenotypes and EMT malignant cell states cooperate to promote tumor progression in esophageal adenocarcinoma. Preprint at Res. Sq. https://doi.org/10.21203/rs.3.rs-2572575/v1.
31. Tian, T., Yang, Z., and Li, X. (2021). Tissue clearing technique: Recent progress and biomedical applications. J. Anat. *238*, 489–507. https://doi.org/10.1111/joa.13309.
32. Dekkers, J.F., Alieva, M., Wellens, L.M., Ariese, H.C.R., Jamieson, P.R., Vonk, A.M., Amatngalim, G.D., Hu, H., Oost, K.C., Snippert, H.J.G., et al. (2019). High-resolution 3D imaging of fixed and cleared organoids. Nat. Protoc. *14*, 1756–1771. https://doi.org/10.1038/s41596-019-0160-8.
33. Messal, H.A., Almagro, J., Zaw Thin, M., Tedeschi, A., Ciccarelli, A., Blackie, L., Anderson, K.I., Miguel-Aliaga, I., van Rheenen, J., and Behrens, A. (2021). Antigen retrieval and clearing for whole-organ immunofluorescence by FLASH. Nat. Protoc. *16*, 239–262. https://doi.org/10.1038/s41596-020-00414-z.
34. Kalluri, R. (2016). The biology and function of fibroblasts in cancer. Nat. Rev. Cancer *16*, 582–598. https://doi.org/10.1038/nrc.2016.73.
35. Lin, M., Hartl, K., Heuberger, J., Beccaceci, G., Berger, H., Li, H., Liu, L., Müllerke, S., Conrad, T., Heymann, F., et al. (2023). Establishment of gastrointestinal assembloids to study the interplay between epithelial crypts and their mesenchymal niche. Nat. Commun. *14*, 3025. https://doi.org/10.1038/s41467-023-38780-3.
36. Qin, X., Sufi, J., Vlckova, P., Kyriakidou, P., Acton, S.E., Li, V.S.W., Nitz, M., and Tape, C.J. (2020). Cell-type-specific signaling networks in heterocellular organoids. Nat. Methods *17*, 335–342. https://doi.org/10.1038/s41592-020-0737-8.
37. Däster, S., Amatruda, N., Calabrese, D., Ivanek, R., Turrini, E., Droeser, R.A., Zajac, P., Fimognari, C., Spagnoli, G.C., Iezzi, G., et al. (2017). Induction of hypoxia and necrosis in multicellular tumor spheroids is associated with resistance to chemotherapy treatment. Oncotarget *8*, 1725–1736. https://doi.org/10.18632/oncotarget.13857.
38. Tang, A., Rappaport, J., Raja, S., Bribriesco, A.C., Sudarshan, M., Siddiqui, H.U., Raymond, D., Murthy, S.C., and Ahmad, U. (2021). Signet Ring Cell Histology Confers Worse Overall Survival in Treated Esophageal Adenocarcinoma. Ann. Thorac. Surg. *111*, 214–222. https://doi.org/10.1016/j.athoracsur.2020.04.134.
39. Mello, A.M., Ngodup, T., Lee, Y., Donahue, K.L., Li, J., Rao, A., Carpenter, E.S., Crawford, H.C., Pasca di Magliano, M., and Lee, K.E. (2022). Hypoxia promotes an inflammatory phenotype of fibroblasts in pancreatic cancer. Oncogenesis *11*, 56. https://doi.org/10.1038/s41389-022-00434-2.
40. Chen, P.Y., Wei, W.F., Wu, H.Z., Fan, L.S., and Wang, W. (2021). Cancer-Associated Fibroblast Heterogeneity: A Factor That Cannot Be Ignored in Immune Microenvironment Remodeling. Front. Immunol. *12*, 671595. https://doi.org/10.3389/fimmu.2021.671595.
41. Mowiol mounting medium (2006). Cold Spring Harbor Protocols. https://doi.org/10.1101/pdb.rec10255.
42. Walters, Z.S., Villarejo-Balcells, B., Olmos, D., Buist, T.W.S., Missiaglia, E., Allen, R., Al-Lazikani, B., Garrett, M.D., Blagg, J., and Shipley, J. (2014). JARID2 is a direct target of the PAX3-FOXO1 fusion protein and inhibits myogenic differentiation of rhabdomyosarcoma cells. Oncogene *33*, 1148–1157. https://doi.org/10.1038/onc.2013.46.

STAR★METHODS

KEY RESOURCES TABLE

REAGENT or RESOURCE	SOURCE	IDENTIFIER
**Antibodies**		
Mouse Anti-Pan-Cytokeratin (Clone AE1/AE3)	Agilent	Cat#M351529-2; RRID:AB_2132885
Rabbit Anti-Vimentin	Cell Signaling Technology	Cat#5741S; RRID:AB_10695459
Rabbit Anti-Cytokeratin 7 (Clone EPR1619Y)	Abcam	Cat#Ab68459; RRID:AB_1139824
Mouse Anti-Cytokeratin 20 (Clone Ks20.8)	Invitrogen	Cat#MA5-13263; RRID:AB_10981940
Rabbit Anti-Ki-67	Abcam	Cat#Ab15580; RRID:AB_443209
Mouse Anti-alpha Smooth Muscle Actin (clone 1A4)	Agilent	Cat#M085101-2; RRID:AB_2223500
Rabbit Anti-Periostin	Abcam	Cat#Ab14041;RRID:AB_2299859
Alexa Fluor 488 Goat Anti-Mouse IgG	Invitrogen	Cat#A-11029; RRID:AB_2534088
Alexa Fluor 633 Goat Anti-Mouse IgG	Invitrogen	Cat#A-21052; RRID:AB_2535719
Alexa Fluor 488 Goat Anti-Rabbit IgG	Invitrogen	Cat#A-11008; RRID:AB_143165
Alexa Fluor 568 Goat Anti-Rabbit IgG	Invitrogen	Cat#A-11036; RRID:AB_10563566
**Biological samples**		
Human esophageal adenocarcinoma FFPE blocks	University Hospital Southampton, Department of Cellular Pathology	See Table 1
**Chemicals, peptides, and recombinant proteins**		
DAPI	Sigma-Aldrich	Cat#D9542-5MG
Hanks' Balanced Salt Solution	ThermoFisher	Cat#14025092
Amphotericin B	ThermoFisher	Cat#15290026
Collagenase P	Sigma-Aldrich	Cat#11213865001
Primocin	InvivoGen	Cat#ant-pm-05
Penicillin-Streptomycin	ThermoFisher	Cat#15140122
Cultrex Basement Membrane Extract, Type 2, Pathclear	Bio-Techne	Cat#3532-005-02
Advanced DMEM/F12	ThermoFisher	Cat#12634010
HEPES	ThermoFisher	Cat#15630056
GlutaMAX	ThermoFisher	Cat#35050061
N-2 Supplement	ThermoFisher	Cat#17502048
B-27 Supplement	ThermoFisher	Cat#17504044
Recombinant Human Noggin	Peprotech	Cat#120-10C-500
Recombinant Human EGF	Peprotech	Cat#AF-100-15-1000
Recombinant Human FGF-10	Peprotech	Cat#100-26-1000
N-Acetylcysteine	Sigma-Aldrich	Cat#A9165-5g
Nicotinamide	Sigma-Aldrich	Cat#N0636-100g
A83-01	TOCRIS	Cat#2939-10mg
SB202190	Stem Cell Technologies	Cat#72634
Y-27632	Sigma-Aldrich	Cat#Y0503-5MG
TrypLE Express	ThermoFisher	Cat#12604013
Fructose	Sigma-Aldrich	Cat#F3510-100G
Glycerol	Sigma-Aldrich	Cat#G5516-500ML
Urea	Sigma-Aldrich	Cat#51456-500G
Zwittergent 3-10	Sigma-Aldrich	Cat#693021-5GM
Boric Acid	Sigma-Aldrich	Cat#B6768
Phalloidin-iFluor594	Abcam	Cat#176757
Triton X-100	Sigma-Aldrich	Cat#T8787-50ML

(*Continued on next page*)

CellPress
OPEN ACCESS

Continued

REAGENT or RESOURCE	SOURCE	IDENTIFIER
Bovine Serum Albumin	Sigma-Aldrich	Cat#A2153-10G
Agarose	Sigma-Aldrich	Cat#A9539-100G
4% Paraformaldehyde	ThermoFisher	Cat#I28800
Xylene, Reagent Grade	Sigma-Aldrich	Cat#214736
Eosin Y solution, Alcoholic	Sigma-Aldrich	Cat#HT110116
Mayer's Hematoxylin	Agilent	Cat#S330930-2
Expert XTF Mounting Medium	CellPath	Cat#SEA-1900-00A
Alcian blue	Sigma-Aldrich	Cat#A3157-10G
Schiff's Reagent	Sigma-Aldrich	Cat#3952016-500ML
Sirius Red F3B	Sigma-Aldrich	Cat#365548-5G
Saturated Aqueous Picric Acid	Sigma-Aldrich	Cat#P6744-1GA
Mowiol 4-88	Sigma-Aldrich	Cat#81381-50G
DABCO 33-LV	Sigma-Aldrich	Cat#290734-100ML
Dulbecco's Modified Eagle's Medium, High Glucose	Sigma-Aldrich	Cat#D6546
Critical commercial assays		
RNeasy Mini Kit	Qiagen	Cat#74104
Deposited data		
Bulk RNA-seq data	This paper	GSE277147
Microscopy data	This paper	https://doi.org/10.5281/zenodo.11639336
Experimental models: Cell lines		
OESO-005	Gift from Dr. Matthew Garnett, Wellcome Sanger Institute, Cambridge, UK	Cell Model Passport ID: HCM-SANG-0290-C15
OESO-191	Gift from Dr. Matthew Garnett, Wellcome Sanger Institute, Cambridge, UK	Cell Model Passport ID: HCM-SANG-0543-C15
OESO-195	Gift from Dr. Matthew Garnett, Wellcome Sanger Institute, Cambridge, UK	Cell Model Passport ID: HCM-SANG-0546-C15
CAF1412	This paper	See Table 1
CAF669	This paper	See Table 1
L-Wnt3A	ATCC	Cat#CRL-2647; RRID:CVCL_0635
HA-R-Spondin1-FC 293T	R&D Systems	Cat#3710-001-01; RRID:CVCL_RU08
Software and algorithms		
Imaris (v9.5.1)	Oxford Instruments	RRID:SCR_007370

EXPERIMENTAL MODEL AND STUDY PARTICIPANT DETAILS

Patient cohort

Patient tumor material was obtained subject to informed consent and local ethical approval (University of Southampton ERGO number 45334) and NHS ethical approval (REC number 18/NE/0234). Key clinical information for each patient involved in organoid and fibroblast derivation is listed in Table 1, along with the relevant organoid identifiers from Cell Model Passports (https://cellmodelpassports.sanger.ac.uk/).

Cell lines

HA-R-Spondin1-Fc 293T cells were obtained from R&D Systems and maintained in DMEM containing 10% fetal calf serum and 300μg/mL Zeocin. After at least five days of selection, cells were expanded to 80% confluence in complete DMEM without Zeocin, and then cultured for another 10 days in Advanced DMEM/F12 with 4mM L-glutamine. Conditioned medium was collected, centrifuged at 3000g for 15 min at 4°C, then sterile filtered and frozen at −80°C.

L-Wnt3A cells (CRL-2647) were obtained from ATCC and maintained in DMEM containing 10% fetal calf serum, 4mM L-glutamine and 400μg/mL Geneticin. Cells were passaged into the desired number of flasks in complete DMEM without Geneticin. Conditioned medium was collected on day 3 and day 7 post-passaging, centrifuged at 3000g for 15 min at 4°C, then pooled, sterile filtered and stored at 4°C for up to 6 months.

METHOD DETAILS

Derivation of patient-derived organoids from EAC tissue

EAC tumor tissue was removed from the resected specimen during esophagectomy using a sterile 6mm biopsy punch. Tumor tissue was placed in a 50mL falcon tube containing 10mL Hanks' Balanced Salt Solution (ThermoFisher) on ice for transport. Tissue was taken into a tissue culture hood and washed three times with PBS containing 250 ng/mL amphotericin B (Invitrogen) (PBSA) for 10 min on ice. Tissue was minced into 1-2mm diameter pieces in digestion buffer with a sterile disposable scalpel: digestion buffer contains EAC-specific organoid media, 30U/mL Collagenase P, 100μg/mL Primocin, 100U/mL penicillin and 100μg/mL streptomycin. Digestion mix was transferred into a 50mL falcon tube and incubated at 37°C with continuous rotation for 1–2 h until tissue was digested and a cloudy suspension was obtained. Every 15 min during the incubation period, the suspension was triturated several times using descending sizes of pipette tip to mechanically break apart the tissue: first 10mL serological pipettes, then 5mL serological pipettes, then 1000μL pipette tips for the last two incubations. The suspension was passed through a 100μm cell strainer and the strainer was washed to collect remaining cells, then centrifuged at 800g for 2 min to pellet. Suspension was washed twice with 30mL PBS and centrifuged to dilute out digestion enzymes. The cell pellet was gently resuspended in 200μL of BME2, deposited as droplets on a single well of a 6-well plate, and allowed to set at 37°C for 15 min. EAC-specific organoid media consisted of advanced DMEM/F12 containing 10mM HEPES, GlutaMAX, *N*-2 and B-27 supplements, 100 ng/ml noggin, 1.25mM N-acetylcysteine, 10mM nicotinamide, 50 ng/mL EGF, 500nM A83-01, 3μM SB202190 and 100 ng/mL FGF-10 as previously described.[15] Conditioned media was collected from L-Wnt3a cells (ATCC CRL-2647) and Cultrex HA-R-Spondin1-Fc 293T cells (R&D Systems), and the final organoid media formulation contained 20% (v/v) R-Spondin1 conditioned media and 50% (v/v) L-Wnt3a conditioned media. 100U/mL penicillin, 100μg/mL streptomycin and 250 ng/mL amphotericin B were added for initial organoid expansion. Once the organoids become crowded or dark, they were passaged by detaching with a pipette, washing with PBS and centrifuging at 800x g for 2 min. The pellet is resuspended in TrypLE for 10 min at 37C and then centrifuged, resuspending in gel containing 320μL BME2 and 80μL cold EAC organoid growth media and deposited as droplets in two wells of a 6-well plate as before. From this point onwards, antibiotic and antimycotic were withdrawn from the organoid growth media.

Derivation of primary fibroblasts from EAC tissue

As above, EAC tumor tissue was removed from the resected specimen during esophagectomy using a sterile 6mm biopsy punch. Tumor tissue was placed in a 50mL falcon tube containing 10mL Hanks' Balanced Salt Solution (ThermoFisher) on ice for transport. Tissue was taken into a tissue culture hood and washed with PBS containing 250 ng/mL amphotericin B (PBSA) for 10 min on ice. Tissue was minced into 1-2mm diameter pieces in a fresh change of PBSA with a sterile disposable scalpel. Using a scalpel, wells of 6-well plates were scratched in an 'X' pattern to create grooves in the plastic, and a single piece of tissue per well was tucked into the middle of the grooves to secure it to the plastic. Complete DMEM containing 10% fetal calf serum, 4mM L-glutamine, 100U/mL penicillin, 100μg/mL streptomycin and 250 ng/mL amphotericin B was gently overlayed and media was changed twice weekly until fibroblasts grew out of the tissue and onto the surrounding plastic. Once this occurred, the tissue piece was gently detached using a 200μL pipette tip. Fibroblasts were gently washed using sterile PBS, detached using TrypLE (ThermoFisher), pooled into a single T25 tissue culture flask, and passaged into larger flasks when 80% confluent. At this point, primary fibroblasts were passaged at a ratio of 1:2-1:3 depending on growth rate and were grown without the addition of amphotericin B to the media.

Production of assembloid models

Assembloid methodology was adapted from pancreatic cancer co-culture methodology previously published by Seino et al.[23] To set up assembloids, a single well of a 6-well plate containing organoids and two T175 flasks of 80% confluent fibroblasts are required. In our experience this takes 2–3 weeks depending on the growth rate of the fibroblasts. Fibroblasts were detached from the flask by washing in PBS and incubating with TrypLE for 10 min, tapping the flask at the end of incubation to facilitate detachment. Fibroblasts were collected by washing in complete DMEM, centrifuging at 800g for 5 min. The fibroblast pellet was resuspended in 5mL of PBS and counted using a hemocytometer. Meanwhile, organoids were collected by pipetting the domes off the well plastic with pre-existing media, washing the well with PBS to collect remaining organoids, pooled and centrifuged at 800g for 2 min. Organoids were resuspended in 5mL of TrypLE and incubated at 37°C for 10–15 min, until organoid structures disaggregated into single cells and small cell clusters. Organoids were centrifuged as before and resuspended in 5mL PBS for counting.

For each model to be made, 2.5×10^4 organoid cells and 5×10^4 fibroblasts were mixed into a single falcon tube. 10% BME2 in ice-cold complete DMEM was prepared, allowing 100μL per model. Cell suspension was centrifuged at 800g for 2 min and the cell pellet was resuspended in 10% BME2. 100μL of cell suspension was dispensed into each well of a Nunclon Sphera low attachment 96-well plate. The plate was centrifuged at 400g for 3 min at room temperature to facilitate cell aggregation, then incubated at 37°C overnight.

The following day, an extracellular matrix (ECM) gel was prepared containing: 10% filter-sterilized 10X DMEM salt solution, 10% fetal calf serum, 60% rat tail collagen I, 20% BME2. 20μL of ECM gel was prepared per model. Using a sterile disposable 3mL Pasteur pipette, co-cultures were picked from each well of the 96-well plate and deposited in a single drop onto tissue culture plastic while minimizing liquid carryover, either into Ibidi 8-well chamber slides or 96-well plates. Excess media around the co-culture was carefully aspirated using a 20μL pipette, and then a single 20μL drop of ECM gel was overlayed onto the co-culture. The tissue culture vessel was inverted to discourage co-culture attachment to the bottom surface and placed in a 37°C incubator for 15 min to allow the ECM

gel to set. Once set, 200µL of prewarmed complete DMEM was gently overlayed. Co-cultures were grown this way for another 7 days, with growth media being changed completely on day 4 of culture.

Wholemount immunofluorescence on assembloid models

Wholemount immunofluorescence methodology was adapted from Dekkers et al.[32] with antigen retrieval methods from the FLASH protocol[33] added to improve antibody penetration and clearing. Co-cultures were fixed for 45 min in 4% paraformaldehyde in PBS at 4°C, then washed twice in cold PBS. Excess ECM gel was removed using 28G needles under a stereomicroscope, an optional step which improves reagent penetration and improves the working distance available for later confocal imaging. Co-cultures were collected into 1.5mL Eppendorf tubes and epitope retrieval was performed using FLASH reagent 2 (250 g/L urea, 80 g/L Zwittergent in 200mM boric acid buffer, pH 7.0) with gentle rotation for 2 h at 37°C. Co-cultures were transferred into 24-well plates, pooling by condition. Co-cultures were washed in organoid washing buffer (OWB) (0.2% BSA and 0.1% Triton X-100 in PBS) at room temperature three times for 10 min, solution was aspirated and primary antibodies diluted in OWB were added to each well (400µL of diluted antibody per well). The plate was incubated for 48 h at 4°C with gentle rocking. Cocultures were then washed briefly three times with OWB, and then washed three more times for: 30 min, 1 h, and 2 h respectively. OWB was completely aspirated, secondary antibodies were diluted in OWB and DAPI was added to 1µg/mL final concentration. Secondary antibody solution was incubated for 48 h at 4°C with gentle rocking.

After repeating the washes as with primary antibody incubations, co-cultures were transferred to fructose-glycerol clearing buffer (60% (v/v) glycerol in 2.5M fructose) for clearing at least 30 min at room temperature prior to imaging. Individual co-cultures were spotted onto a glass coverslip with a drop of fructose-glycerol clearing buffer and mounted for imaging in a Leica SP8 laser-scanning confocal microscope. Optical sections were acquired until intensity of DAPI nuclear stain tailed off, normally at ~200-250µm z-depth. Image files were processed in Imaris v9.5.1 (Oxford Instruments) and rendered in blend mode. Orthogonal projections, maximum intensity z-projections and movies were generated using the same software (Videos S1, S2 and S3).

Agarose embedding of organoids and assembloids for paraffin histology

Organoids in monoculture and co-culture were grown as described above. For monocultures, organoids were collected by pipetting up and down gently to dissociate the BME2 and collected into a 1.5mL microcentrifuge tube. For co-cultures, specimens were washed and fixed *in situ* on the tissue culture plastic, either on µ-Slide 8-well chamber slides (ibidi) or in 96-well plates. In both cases, cultures were gently washed with ice-cold PBS, then fixed in 4% paraformaldehyde (PFA) for 45 min at 4°C. Monoculture organoids were allowed to settle naturally at the bottom of the tube during washes before gently aspirating supernatant.

To handle organoids more easily during paraffin embedding, organoids and co-cultures were pre-embedded in 2% (w/v) agarose. PFA-fixed organoids and co-cultures kept in PBS were allowed to settle to the bottom of the tube and equilibrate to 60°C in a hot block, supernatant was removed, and then 500uL agarose solution pre-equilibrated to 60°C was added. Tubes were placed in a hot block set to 60°C to allow specimens to sink for 10 min, and then placed on ice for 5 min to solidify the agarose. Agarose blocks were removed from the tubes, trimmed with a scalpel, and placed in 70% ethanol for at least 1 h prior to processing for paraffin embedding using standard histological protocols.

Hematoxylin & eosin staining

FFPE tissue sections of 4µm thickness were deparaffinized, rehydrated, stained with Mayer's hematoxylin for 5 min, and blued in running tap water. Sections were counterstained with Eosin Y for 5 min, rinsed briefly in distilled water, then dehydrated, cleared and mounted in resinous mounting medium. For imaging, slides were scanned using an LM dotSlide (Olympus) with a 20x magnification objective. Scanned H&E stains were reviewed digitally by a specialist gastrointestinal pathologist blinded to specimen identity to assess histological differentiation in both patient tumor tissue and in co-culture specimens.

Alcian blue – Periodic acid schiff staining

FFPE tissue sections of 4µm thickness were deparaffinized, rehydrated, and stained in Alcian blue (1% Alcian blue (w/v) in 1% (v/v) aqueous acetic acid). Sections were washed in running tap water for 2 min, briefly rinsed in distilled water, treated with 0.5% (w/v) aqueous periodic acid for 5 min, rinsed again in distilled water, and then stained with Schiff's reagent for 10 min. Sections were counterstained with Mayer's hematoxylin for 2 min, blued in tap water, dehydrated, cleared and mounted in resinous mounting medium. For imaging, slides were scanned using an Olympus LM dotSlide with a 20x magnification objective.

Picro-sirius red staining

FFPE tissue sections of 4µm thickness were deparaffinized, rehydrated, stained with Mayer's hematoxylin for 2 min, then stained with 0.1% (w/v) Sirius Red F3B in saturated aqueous picric acid for 1 h. Sections were rinsed for 30 s in 0.01% (v/v) HCl, rinsed in distilled water, dehydrated, cleared and mounted in resinous mounting medium. For imaging, slides were scanned using an Olympus LM dot-Slide with a 10x magnification objective and an analyzer and polarizing filter inserted.

Double immunofluorescence on FFPE embedded tissue and organoids

FFPE tissue sections of 4μm thickness were deparaffinized and rehydrated in a graded ethanol series. Heat-induced epitope retrieval was performed by microwaving on 50% power for 25 min in sodium citrate buffer (10mM sodium citrate, 0.05% Tween 20, pH 6.0) or Tris-EDTA buffer (10mM Tris base, 1mM EDTA, 0.05% Tween 20, pH 9.0) depending on the antibodies to be used. Sections were blocked in 2.5% normal horse serum for 30 min and then incubated with primary antibodies at pre-determined dilutions diluted in antibody buffer (5% bovine serum albumin in TBS with 0.05% Tween 20). Sections were then washed three times, and secondary antibodies diluted in antibody buffer containing 1μg/mL DAPI were applied for 1 h in the dark. Sections were washed three times and mounted with Mowiol mounting medium,[41] cured overnight at room temperature prior to imaging. Representative images of specimens were acquired using a Leica SP8 laser-scanning confocal microscope.

qRT-PCR of CAFs

Total RNA from CAFs was isolated using the RNeasy Mini Kit (Qiagen) according to manufacturer's instructions. cDNA was synthesized using SuperScript II (Invitrogen) and qRT-PCR was performed as reported previously[42] using QuantStudio 7 Flex (Applied Biosystems). TaqMan primer and probe sets for EPCAM, POSTN, ACTA2, GSN, COL1A1, CXCL8, CXCL14 and TRPA1 were from Applied Biosystems.

Bulk RNA-sequencing of organoids, CAFs and assembloids

Total RNA from organoids, CAFs and assembloids was isolated using the RNeasy Mini Kit (Qiagen) according to manufacturer's instructions. mRNA library preparation and sequencing was outsourced to Novogene using an Illumina 150bp paired-end sequencing strategy. The raw fastq data for a total of 8 samples (2 CAFs, 3 Organoid and 3 assembloid co-cultures) were quality-checked using fastQC (v0.12.1) and aligned to the reference genome (GRCh38) using Star aligner (V2.7.10b), all the samples passed quality checks and had at least 80 percent of uniquely mapped reads. The count matrix was generated using Htseq-count (V2.0.3). The top 2000 most variable genes (based on standard deviation) were clustered (k-means) and seven clusters of genes that were differentially expressed between samples were identified and plot using R package complexHeatmap (v2.16.0). R package ggplot2 (v3.5.1) was used to plot principal component analysis (PCA) based on the top two principal components, and to plot the TPM per CAF for a selection of genes based on known CAF markers. A hypoxia score was calculated for each organoid and assembloid based on the mean TPM of 200 genes upregulated in hypoxia (from hallmark hypoxia gene set). Additional R packages used to display data included dplyr (v1.1.4), ggpubr (v0.6.0), reshape2 (v1.4.4), and stringr (v1.5.1).

QUANTIFICATION AND STATISTICAL ANALYSIS

For comparisons of signature scores from bulk RNA-seq data (Figure S3), statistical analysis was performed with R (v4.2.0) and n is defined in the corresponding figure legend. T-tests were used to compare groups. $p < 0.05$ was considered statistically significant (p-value is stated above the comparison). The top, middle and bottom line of the boxplot represents the upper quartile (Q3), median, and lower quartile (Q1) respectively. The maximum value represented by the top whisker represents the highest observed data point within Q3 + (1.5 x (Q3-Q1)), and the minimum value represented by the bottom whisker represents the lowest situated point within Q1 - (1.5 x (Q3-Q1)) (Figure S3B). All error bars represent standard deviation (Figure S2B).

Cell Reports Methods, Volume *4*

Supplemental information

Patient-derived tumor organoid and fibroblast assembloid models for interrogation of the tumor microenvironment in esophageal adenocarcinoma

Benjamin P. Sharpe, Liliya A. Nazlamova, Carmen Tse, David A. Johnston, Jaya Thomas, Rhianna Blyth, Oliver J. Pickering, Ben Grace, Jack Harrington, Rushda Rajak, Matthew Rose-Zerilli, Zoe S. Walters, and Tim J. Underwood

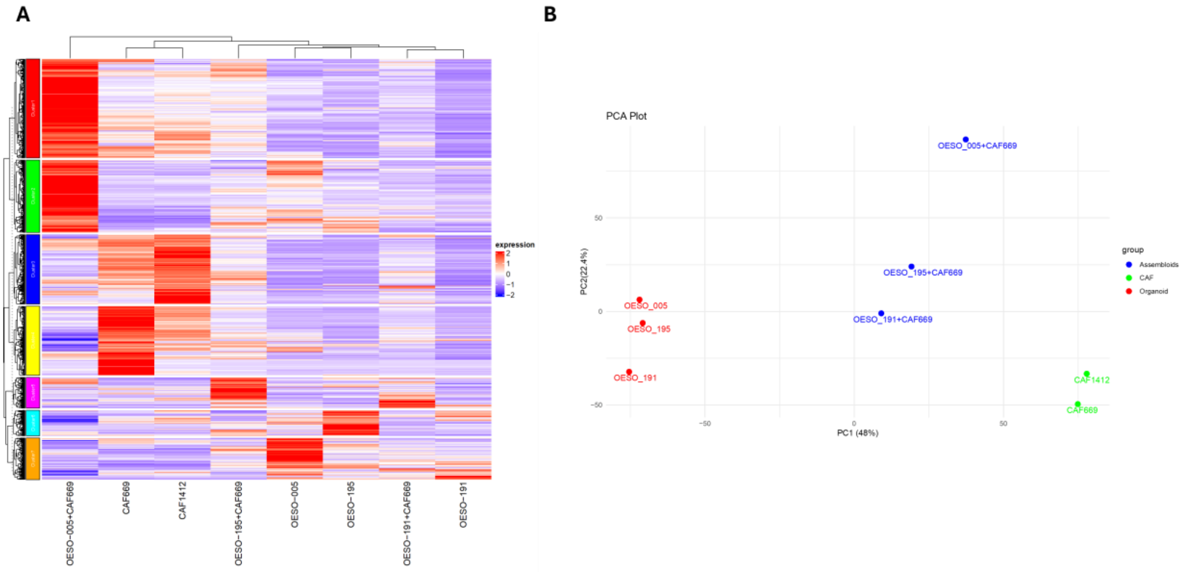

Figure S1. Descriptive analysis of RNA-seq data from assembloids and corresponding organoids and fibroblasts, related to Figure 2.

A.) To identify differences/similarities between assembloids and constituent components (organoids and CAFS), we performed cluster analysis on the top 2000 variable genes in the Transcript Per Millions count matrix to explore differences between samples. This analysis clusters the two CAF together and identified 7 distinct gene clusters, each showing unique gene expression patterns compared to the others. B.) To understand the distribution of samples, we conducted a PCA analysis revealing similarity across similar groups.

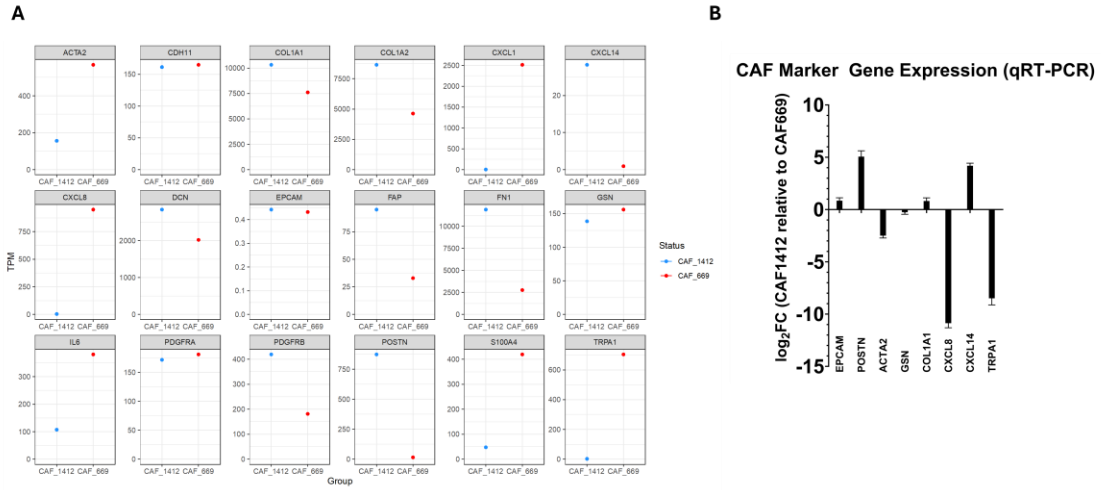

Figure S2. Phenotyping of CAF cultures by RNA-seq and qRT-PCR, related to Figure 2.

A.) Analysis of key myofibroblast and inflammatory CAF markers from bulk RNAseq data shows that both CAFs express key markers associated with myoCAF identity. CAF669 also expresses markers associated with a more proinflammatory CAF phenotype (IL6). B.) However, qRT-PCR analysis of key markers showed that the two CAFs were more similar in expression of myoCAF markers rather than in iCAF markers.

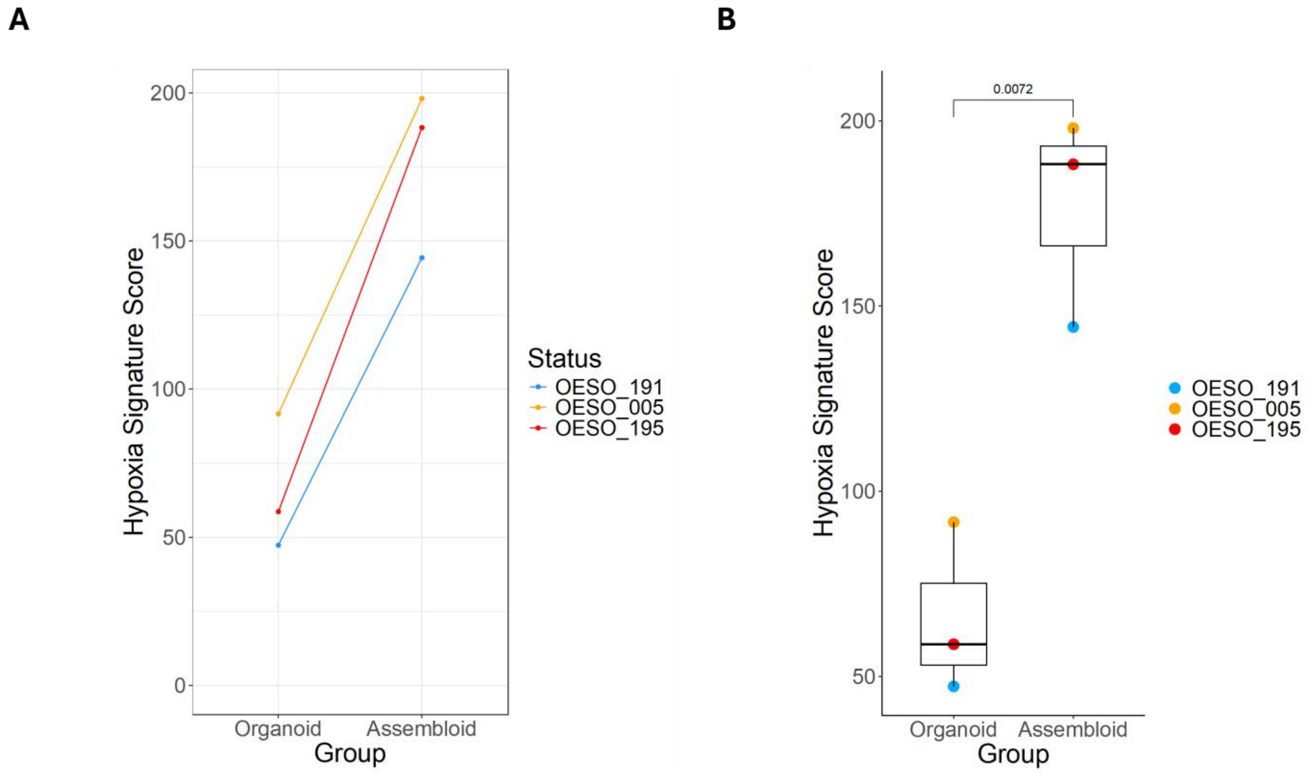

Figure S3. Comparison of hypoxia-related gene expression in organoids and corresponding assembloids, related to Figure 2.

A.) To identify whether hypoxia is a feature of co-cultures compared to organoids alone, we used the 200 genes in the hallmark hypoxia pathway to score each sample. B.) Analysis of bulk RNAseq data (n=3 sequenced samples per group) shows that in all cases the hypoxia signature is higher in co-culture, and when taken together the difference is significant (t-test).
